# Laparoscopic Sleeve Gastrectomy in Patients with Severe Obesity Restores Adaptive Responses Leading to Nonalcoholic Steatohepatitis

**DOI:** 10.3390/ijms23147830

**Published:** 2022-07-15

**Authors:** Noemí Cabré, Fedra Luciano-Mateo, Douglas J. Chapski, Gerard Baiges-Gaya, Salvador Fernández-Arroyo, Anna Hernández-Aguilera, Helena Castañé, Elisabet Rodríguez-Tomàs, Marta París, Fàtima Sabench, Daniel Del Castillo, Josep M. del Bas, Mercedes Tomé, Clément Bodineau, Alejandro Sola-García, José López-Miranda, Alejandro Martín-Montalvo, Raúl V. Durán, Thomas M. Vondriska, Manuel Rosa-Garrido, Jordi Camps, Javier A. Menéndez, Jorge Joven

**Affiliations:** 1 Unitat de Recerca Biomèdica, Department of Surgery, Institut d’Investigació Sanitària Pere Virgili, Hospital Universitari de Sant Joan, Universitat Rovira i Vigili, 43204 Reus, Spain; noemi.cabre@gmail.com (N.C.); fedra.luciano@gmail.com (F.L.-M.); gerard.baiges@iispv.cat (G.B.-G.); salvador.fernandezarroyo@gmail.com (S.F.-A.); anna.hernandeza@gmail.com (A.H.-A.); helena.castanev@gmail.com (H.C.); elisabet.rodriguez@urv.cat (E.R.-T.); marta.paris@salutsantjoan.cat (M.P.); fatima.sabench@urv.cat (F.S.); ddcasdej@gmail.com (D.D.C.); 2 Department of Anesthesiology, David Geffen School of Medicine, University of California, Los Angeles, CA 90095, USA; dchapski@ucla.edu (D.J.C.); tvondriska@mednet.ucla.edu (T.M.V.); 3 Technological Unit of Nutrition and Health, EURECAT Technology Center of Catalonia, 43204 Reus, Spain; josep.delbas@eurecat.org; 4 Centro Andaluz de Biología Molecular y Medicina Regenerativa, CSIC, University of Sevilla, 41004 Sevilla, Spain; mercedes.tome@cabimer.es (M.T.); cbodineau@bwh.harvard.edu (C.B.); alejandro.sola@cabimer.es (A.S.-G.); alejandro.martinmontalvo@cabimer.es (A.M.-M.); raul.duran@cabimer.es (R.V.D.); 5 Lipids and Atherosclerosis Unit, IMIBIC/Reina Sofía University Hospital, CIBER Fisiopatología de la Obesidad y Nutrición, ISCIII, 28029 Cordoba, Spain; jlopezmir@uco.es; 6 Department of Biomedical Engineering, University of Alabama, Birmingham, AL 35233, USA; mrgarrido@uab.edu; 7 Girona Biomedical Center and Program against Cancer Therapeutic Resistance, Catalan Institute of Oncology, 08908 Barcelona, Spain; jmenendez@idibgi.org

**Keywords:** bariatric surgery, DNA methylation, energy metabolism, epigenetics, functional studies, glutaminolysis, multi-omics approach

## Abstract

The surgically induced remission of liver disease represents a model to investigate the signalling processes that trigger the development of nonalcoholic steatohepatitis with the aim of identifying novel therapeutic targets. We recruited patients with severe obesity with or without nonalcoholic steatohepatitis and obtained liver and plasma samples before and after laparoscopic sleeve gastrectomy for immunoblotting, immunocytochemical, metabolomic, transcriptomic and epigenetic analyses. Functional studies were performed in HepG2 cells and primary hepatocytes. Surgery was associated with a decrease in the inflammatory response and revealed the role of mitogen-activated protein kinases. Nonalcoholic steatohepatitis was associated with an increased glutaminolysis-induced production of α-ketoglutarate and the hyperactivation of mammalian target of rapamycin complex 1. These changes were crucial for adenosine monophosphate-activated protein kinase/mammalian target of rapamycin-driven pathways that modulated hepatocyte survival by coordinating apoptosis and autophagy and affected methylation-related epigenomic remodelling enzymes. Hepatic transcriptome signatures and differentially methylated genomic regions distinguished patients with and without steatohepatitis. Our results suggest that the increased glutaminolysis-induced α-ketoglutarate production and the mammalian target of rapamycin complex 1 dysregulation play a crucial role in the inefficient adaptive responses leading to steatohepatitis in obesity.

## 1. Introduction

Predictions regarding the epidemic of obesity indicate an exponential increase in the incidence of nonalcoholic steatohepatitis (NASH), which has become a major risk factor for liver disease-associated deaths [1]. The mechanisms that lead to NASH onset and progression involve the complex effects of multiple regulatory pathways and adaptive responses [2] that may be initially protective but eventual failures result in mitochondrial dysfunction and hepatocellular death [3]. The sensors and controllers of energy storage and expenditure, combined with the efficient regeneration ability and the functional reserve of the liver, are likely regulators of the stress responses that may lead to chronic liver disease or may contribute to NASH remission. Consequently, the pathways that are driven by the relationship between AMP-activated protein kinase (AMPK) and the mammalian target of rapamycin complex 1 (mTORC1) may play a key role in metabolic damage, cell survival, and programmed cell death control [4].

Metabolomic studies of NASH remission might offer a unique opportunity to examine the signalling processes that govern the alterations in the liver architecture, the self-perpetuating cycle of oxidative and inflammatory stresses, and the hepatocellular death that characterises NASH [5,6]. We used quantitative targeted metabolomics [7] for the measurement of the concentrations of metabolites from energy and one-carbon (1-C) metabolism pathways in the liver by defining a set of metabolites that could facilitate the assessment of the interconnected pathways that are crucial for mitochondrial processes and the regulation of metabolic reprogramming [8]. These metabolites are also signalling molecules that affect gene expression and chromatin structure and play a direct role in methylation reactions [9] that might explain the emerging relationship between metabolism, methylation, and hepatic steatosis [10]. Here, we provide evidence supporting the multifaceted and interrelated potential of the glutaminolysis-derived production of hepatic α-ketoglutarate (α-KG) and the deregulation of mTORC1 signalling to promote NASH.

## 2. Results

### 2.1. NASH Remission Included the Reversal of the Altered Liver Mitochondrial Status and Oxidative Stress 

**Figure 1:**
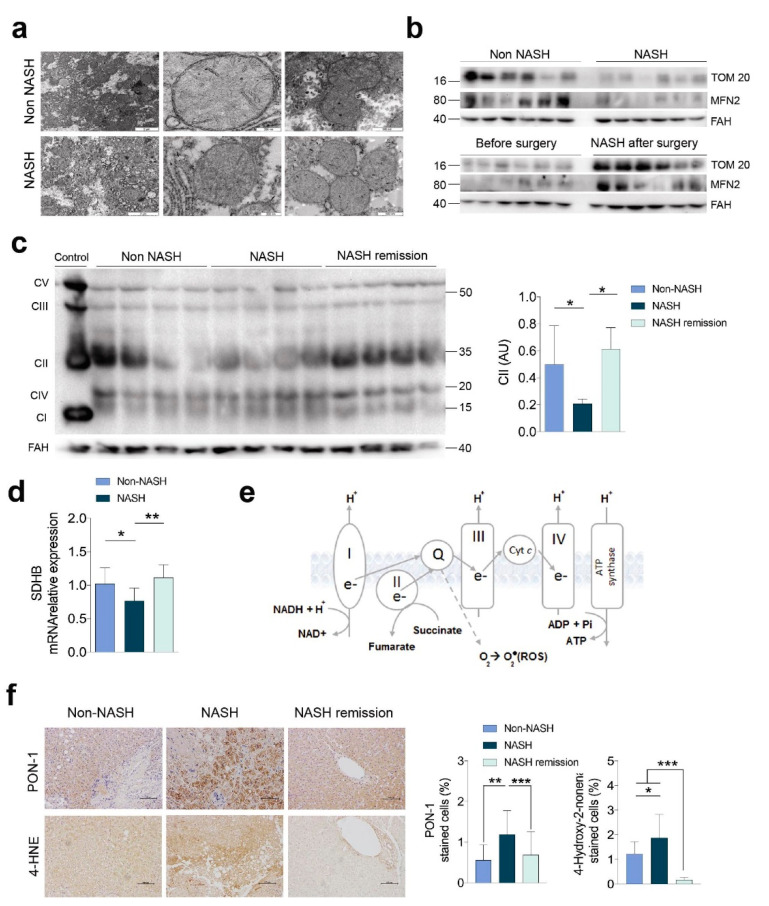
Impairment of oxidative phosphorylation and oxidative stress were reversible in the livers of patients with NASH: (**a**) transmission electron microscopy of hepatocyte mitochondria; (**b**) representative Western blots of Tom20 and Mfn2 (n = 12, each group); (**c**) representative Western blots of the OXPHOS complexes; (**d**) mRNA expression of succinate dehydrogenase B recapitulated the changes in complex II (n = 12, each group); (**e**) a schematic figure representing the importance of complex II in oxidative phosphorylation; (**f**) alterations in 4-hydroxy-2-nonenal and paraoxonase-1 (PON-1) (n = 31, each group). * *p* < 0.05, ** *p* < 0.01, and *** *p* < 0.001 by Mann–Whitney *U* test. In panel (**c**), the term “control” refers to a positive technical control. In the rest of the experiments, the results of patients with NASH were compared with those of patients without NASH and those after surgery with those before. In panel b, the non-NASH/NASH and before/after surgery blot pairs are shown separately, because they were run on different gels. The results are shown as the means and SD. The numbers to the left of the blots in panel b and to the right in panel c are the molecular weights. FAH: Fumarylacetoacetate hydrolase.

Bariatric surgery successfully resolved NASH and mitigated the metabolic comorbidities. Steatosis and hepatocyte ballooning were no longer evident, and fibrosis and lobular inflammation were significantly improved (Table S1). The differences in the microscopic mitochondrial morphology of hepatocytes suggested defective mitochondrial biogenesis and defective removal of damaged mitochondria in NASH. Mitochondria in the livers of patients with NASH were smaller and with a more heterogeneous shape, indicating an apparent decrease in fusion in favour of fission (Figure 1a). The significant reduction in the expression of mitofusin 2 (MFN2) and translocase of the outer membrane 20 (TOM20) in the livers of patients with NASH also suggested that the number of mitochondria was lower, and they had defects in fusion. The recovery of the expression of these molecules was complete after NASH remission (Figure 1b and Figure S1a). Changes in the expression of the respiratory complexes and the likely impairment of oxidative phosphorylation (OXPHOS) characterised NASH (Figure 1c–e and Figure S1b). The most significant findings were observed in complex II (i.e., succinate dehydrogenase), which exhibited reduced expression in the livers of patients with NASH and complete recovery after NASH remission. These differences were paralleled by changes in the mRNA transcription of succinate dehydrogenase B (SDHB), which is the subunit that participates in the electron transport chain [11]. Surrogates for the increased generation of ROS and antioxidant enzymes, such as 4-hydroxy-2-nonenal and paraoxonase-1, were sufficient to unambiguously distinguish the livers from patients with and without NASH (Figure 1f). The reversal of the altered mitochondrial status and the recovery of the disrupted signalling pathways resulted in benefits to tissue cells that likely participate in NASH remission.

### 2.2. NASH Reflects an Impact on Multiple Stress-Responsive Pathways

**Figure 2:**
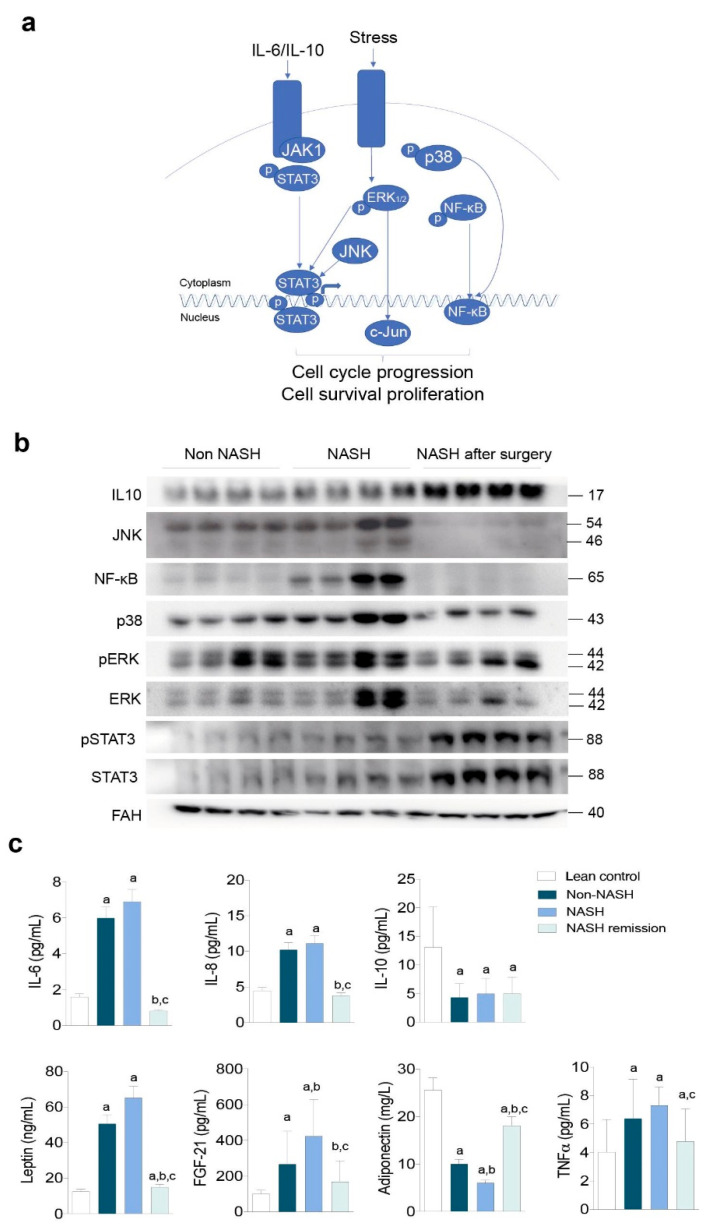
Adaptive responses to hepatocellular damage and oxidative and inflammatory stresses determined the severity of liver disease: (**a**) schematic representation of the regulatory role of MAPKs; (**b**) representative Western blots indicating that NASH remission reversed the molecular signals associated with mitochondrial stress but increased IL-10 expression and STAT-3 activation (n = 16, each group); (**c**) circulating cytokine concentrations in patients and lean controls. ^a^ At least *p* < 0.05 with respect to the controls. ^b,c^ At least *p* < 0.05 compared to the livers of patients without and with NASH, respectively, by the Mann–Whitney *U* test. In panel 1c, the term “lean control” refers to a lean individual. The results are shown as the means and SD. The numbers to the right of the blots are the molecular weights. FAH: Fumarylacetoacetate hydrolase.

The association of oxidative and inflammatory responses with hepatocellular damage suggests the regulatory role of mitogen-activated protein kinases (MAPKs; Figure 2a). We then explored the nuclear factor-κB (NF-κB) signalling pathway, which responds to mitochondrial dysfunction and affects cytokine expression and release. The hepatic expression of NF-κB and p38 was higher in the livers of patients with NASH than in the livers of patients without NASH and decreased after NASH remission (Figure 2b and Figure S1c). The same response was observed in the expression of c-Jun amino-terminal kinase (JNK), which was dependent on mitochondrial dysfunction, and in the expression and phosphorylation of extracellular signal-regulated kinases (ERKs), which suggested the reversibility of the changes in the endoplasmic reticulum (Figure 2b). The expression of interleukin (IL)-10 and the activation of the signal transducer and activator of transcription-3 (STAT-3) signalling were similar in patients with and without NASH. Conversely, we found a simultaneous increase in the expression of these molecules after NASH resolution that was apparently linked to tissue repair and favourable adaptive responses (Figure 2b). In the context of obesity, the hepatic mitochondrial stress-related adaptive functions of STAT-3 signalling appear to be dissociated from the canonical response to cytokine imbalance (Figure 2a,b). We then explored the release of cytokines into circulation and found that the changes likely resulted from the synchronised activation of different inflammatory signals in other organs (Figure 2c). The circulating levels of IL-6, IL-8, and tumour necrosis factor alpha (TNF-α) distinguished patients with obesity and lean controls but did not distinguish patients with and without NASH (Figure 2c). NASH remission was associated with significant weight loss, though patients were still obese (mean BMI = 31.4 Kg/m^2^ one year after surgery). Circulating IL-10 levels did not change after NASH remission (Figure 2b,c). The concentrations in blood of leptin, fibroblast growth factor-21 (FGF-21), and adiponectin normalised after NASH remission and were apparently associated with liver damage suggesting the importance of inter-organ crosstalk in NASH progression (Figure 2c). Interestingly, cytokines are accepted molecular mediators of apoptosis and might be associated with the clearance of dysfunctional cells.

### 2.3. NASH Remission Reversed Apoptosis and Reactivated Autophagy via mTORC1 Signalling

**Figure 3:**
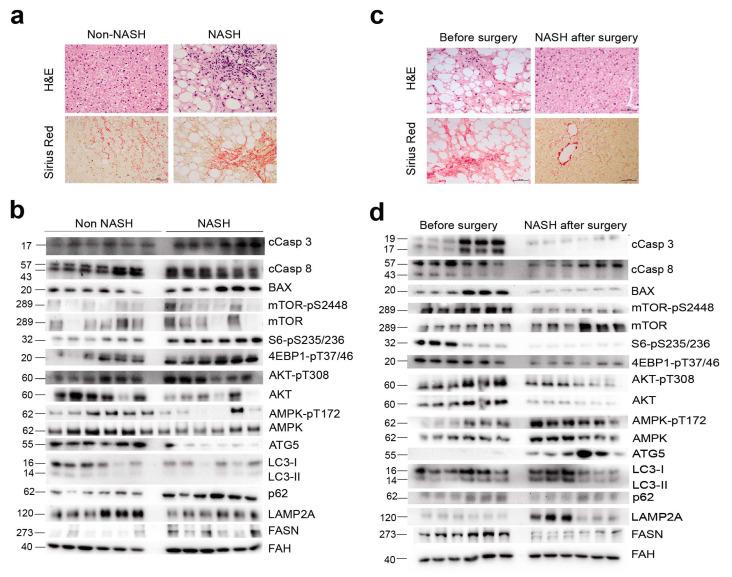
Hepatic AMPK/mTOR-driven pathways coordinated apoptosis and autophagy in liver disease: (**a**) histology differentiated the livers of patients with and without NASH and (**b**) confirmed the cellular improvement after NASH remission; (**c**) representative Western blots comparing selected markers in the livers of patients with and without NASH (n = 18, each) indicated increased apoptosis and compromised autophagy in NASH, accompanied by decreased AMP phosphorylation and increased mTOR phosphorylation; (**d**) the same markers examined in patients with NASH before and after surgery (n = 18, each) indicated that NASH remission reversed apoptosis and reactivated autophagy. In panels b and d, the non-NASH/NASH and before/after surgery blot pairs are shown separately, because they were run on different gels. The numbers to the left of the blots are the molecular weights. FAH: Fumarylacetoacetate hydrolase.

Histological damage was mitigated after NASH remission (Figure 3a,b), and the measurement of apoptotic nuclei by terminal deoxynucleotidyl transferase dUTP nick end labelling confirmed the increased apoptosis in NASH livers and its association with disease severity. Indeed, values were higher in patients with fibrosis, decreased after surgery, and correlated with steatosis (Figure S2), although the correlation between apoptotic nuclei and steatosis suggested two distinct populations, one with high steatosis and another with low steatosis, with a significant overlap of apoptotic nuclei. Pro-apoptotic markers, including the release and cleavage of caspases and the expression of B-cell lymphoma 2-associated X protein, were significantly higher in the livers of patients with NASH than in the livers of patients without NASH (Figure 3c and Figure S3a). Increased apoptosis was accompanied by compromised autophagy and chaperone-mediated autophagy (CMA), as indicated by an altered LC3 II to LC3 I ratio, the accumulation of p62, and the reduced expression of the CMA-limiting component lysosome-associated membrane protein type 2A (LAMP2A).

The balance between apoptosis and autophagy appeared to be coordinated. The decreased phosphorylation of AMPK with the increased expression of fatty acid synthase (FASN) and the increased phosphorylation of mTOR, S6, 4E-BP1, and AKT (Figure 3c) suggested a key regulatory role of mTORC1 signalling in apoptosis induction and autophagy inhibition in patients with NASH. Livers after NASH remission exhibited lower FASN expression; increased AMPK phosphorylation; decreased mTOR, S6, 4E-BP1, and AKT phosphorylation. These changes and the lack of p62 accumulation, the normalised levels of LC3II and LC3I, and the increased expression of LAMP2A suggest the inhibition of apoptosis and the restoration of hepatic autophagy and CMA downstream of mTORC1 (Figure 3d and Figure S3b). Our data suggest that hepatic mTORC1 hyperactivation perturbs the harmonization between autophagy and apoptosis. It may be argued that the AMPK/mTOR signalling cascade is important in the stress and metabolic responses that appear to promote NASH.

### 2.4. Metabolomics Revealed the Role of Glutaminolysis Activation in NASH Metabolic Rewiring

**Figure 4:**
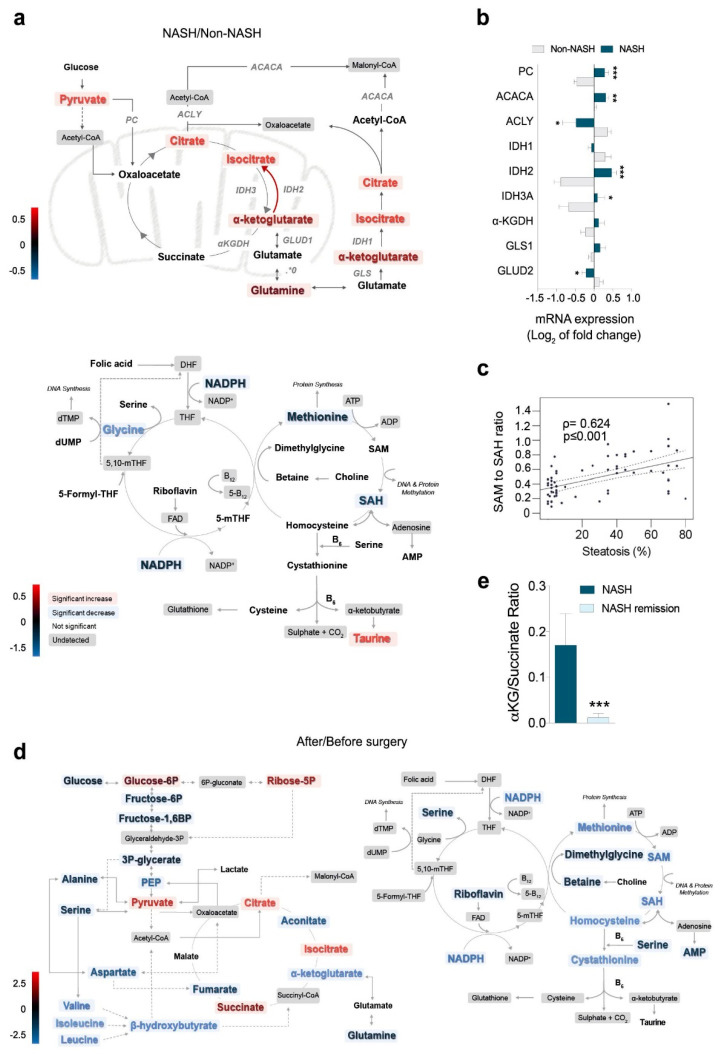
The liver metabolome revealed the key role of glutaminolysis activation in NASH: (**a**) the accumulation of glutamine, α-KG, citrate, and pyruvate in the livers of patients with NASH was the most prominent metabolomic finding; (**b**) significant mRNA overexpression of glutamate dehydrogenase, glutaminase, and isocitrate dehydrogenases and underexpression of α-KG-dehydrogenase and pyruvate carboxylase in the livers of patients with NASH; (**c**) changes in the methionine cycle; (**d**) NASH remission increased the entry of glucose carbon into the mitochondria and decreased anaplerotic reactions; (**e**) NASH remission significantly decreased the α-KG-to-succinate ratio. * *p* < 0.05, ** *p* < 0.01, and *** *p* < 0.001, by the Mann–Whitney *U* test. The results are shown as the means and SD. The red colour indicates an increase and the blue colour a decrease in metabolite concentrations comparing the groups indicated in the figure. Colour intensity indicates the.

Metabolites from energy and 1-C metabolism distinguished patients with obesity and with and without NASH (Figure S4a), and the more prominent differences were found in α-ketoglutarate (α-KG) to succinate conversion and in glutaminolysis (Table S2). The concentrations of glutamine, α-KG, citrate, and pyruvate were higher in patients with NASH than in patients without NASH, suggesting an impairment of the mitochondrial citric acid cycle (CAC) (Figure 4a). The changes in metabolites were associated with significant differences in the hepatic mRNA transcription of the involved enzymes. Glutamate dehydrogenase, glutaminase (GLS1), and isocitrate dehydrogenases were overexpressed, and α-KG dehydrogenase (α-KGDH) and pyruvate carboxylase were underexpressed in NASH (Figure 4b). These results suggested the activation of glutaminolysis as the source of the increased α-KG levels in patients with NASH. The concentrations of metabolites from 1-C metabolism were also altered in these patients including significantly decreased concentrations of glycine, SAH, and methionine. The SAM-to-SAH ratio correlated with steatosis (Figure 4c). After NASH remission, the metabolic differences were profound and clearly distinguished paired liver specimens (Figure S4b, Table S2). The reduced hepatic levels of glucose and glycolytic intermediates distal to glucose-6-phosphate, hydroxybutyrate, and amino acids indicated the increased entry of glucose carbon into mitochondrial biosynthetic metabolism and the improvement of glycolysis and insulin sensitivity after NASH remission. The metabolites from 1-C metabolism remained unaltered or significantly decreased after NASH remission, but the SAM-to-SAH ratio was completely restored (Figure 4d). The high α-KG-to-succinate ratio observed in the livers of patients with NASH was reversed after NASH remission (Figure 4e) as a consequence of decreased anaplerotic reactions that drive glutamine-derived carbon into the mitochondria. Metabolic changes in plasma are very similar to those in the liver (Figure S5a,b). Our results suggest, therefore, that glutamine was favoured as a source of energy in patients with NASH accompanied by perturbations in the methionine cycle, glutaminolysis, and α-KG to succinate conversion. The findings confirmed in the liver the mutual dependence between mTORC1 and glutamoptosis [12].

### 2.5. Increased α-Ketoglutarate Altered Mitochondrial Metabolism and Apoptosis in Cultured Hepatocytes

**Figure 5:**
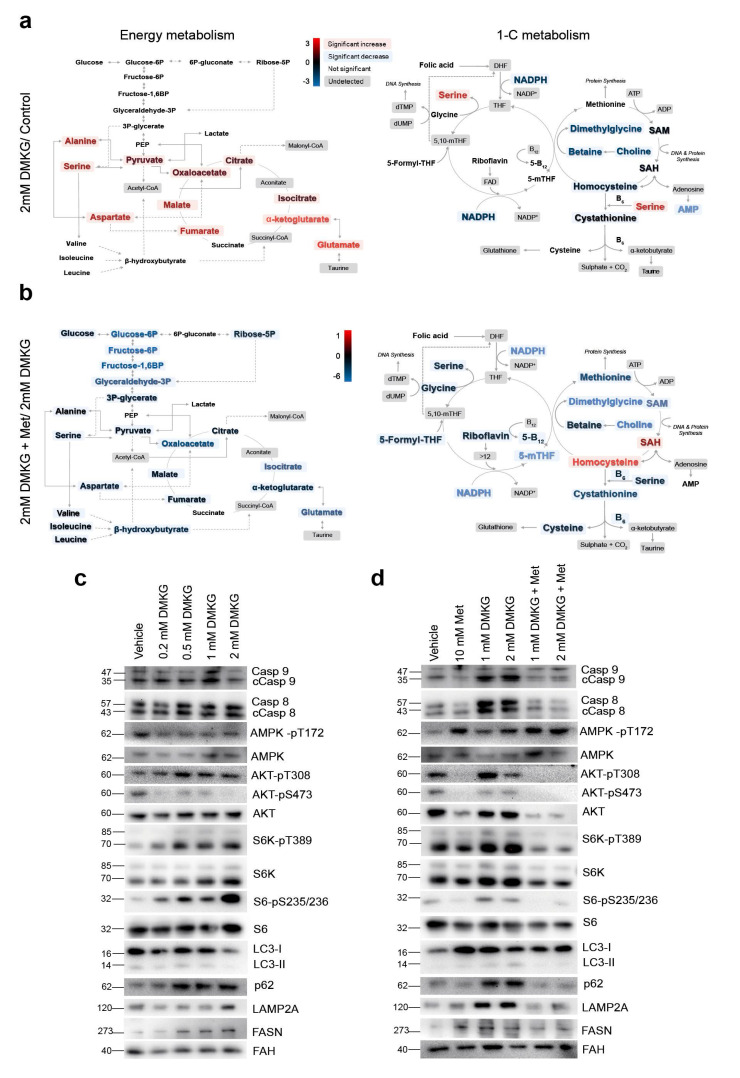
In cultured hepatocytes, increases in cellular α-KG altered mitochondrial metabolism, apoptosis, and autophagy. (**a**) Quantitative metabolomics revealed that the increased α-KG levels altered the amino acid metabolome and resulted in the accumulation of metabolites from the citric acid cycle. Metabolites from one-carbon metabolism were either unaffected or significantly decreased. (**b**) Supplementation with metformin abrogated most of the α-KG-induced metabolic effects. (**c**) Representative Western blots of selected markers indicated a dose-dependent effect of α-KG in stimulating apoptosis and decreasing autophagy via mTORC1 activation. (**d**) Metformin also abrogated these effects by increasing AMPK phosphorylation. “Vehicle” blots are blots without DMKG and were used as controls. The red colour indicates an increase and the blue colour a decrease in metabolite concentrations comparing the groups indicated in the figure. Colour intensity indicates the degree of change, expressed as log2 fold-change, according to the scale shown next to the panels. The numbers to the left of the blots are the molecular weights. FAH: Fumarylacetoacetate hydrolase.

The causality of our findings, although suggestive, is difficult to infer with this design. We then examined whether these metabolic changes influenced apoptosis and autophagy in HepG2 cells by supplementing a cell-permeable αKG derivative (dimethyl 2-oxoglutarate, DMKG) (Figure S6a). Even moderate increases in the cellular α-KG levels resulted in dose-dependent, progressive changes in the metabolites from energy and 1-C metabolism, and distinct metabolic profiles were observed in the supplemented cells compared to the untreated cells (Table S3, Figure S6b). The altered amino acid metabolome resulted in increased alanine and serine levels, likely as a consequence of the altered metabolism of pyruvate and glycine, and no changes in glycolysis were observed. Treatment with 2 mM DMKG increased the α-KG-to-succinate ratio and accumulated metabolites from the CAC but resulted in either unaffected or significantly decreased concentrations of metabolites from 1-C metabolism (Figure 5a). Hence, the addition of α-KG recapitulated the metabolic changes observed in NASH livers. As expected [13], supplementation with metformin abrogated the α-KG-induced metabolic effects. The alterations in the amino acid metabolome were reversed, the concentration of the metabolites from glycolysis and the CAC decreased, and changes in the methionine cycle resulted in the reversal of the SAM-to-SAH ratio (Figure S6c and Figure 5b). The increased intracellular α-KG levels increased cell death in a dose-dependent manner (Figure S7a). Flow cytometry analysis of annexin V- and propidium iodide-stained cells revealed the impact of high α-KG levels on the intrinsic effects of metformin (Figure S7b,c and Figure 5c). The progressive accumulation of p62 and the decreased formation of LC3II suggested a combination of increased apoptosis and compromised autophagy in this model (Figure 5c). The mechanisms were likely related to mTORC1 activation as indicated by the decrease in the phosphorylation of AMPK and the increase in phosphorylation of S6 and AKT at T308. The lack of AKT phosphorylation at S473 suggested a negligible role for mTORC2, which correlated with nonsignificant changes in the expression of LAMP2A and FASN (Figure 5c). The AMPK-dependent inhibition of mTORC1 by metformin was demonstrated by the increase in AMPK phosphorylation and the consequent decrease in S6 phosphorylation, which was accompanied by increased degradation of p62, decreased levels of cleaved caspases and LAMP2A and altered levels of LC3II (Figure 5d). We re-examined these findings using primary hepatocytes within an ex vivo approach in which α-KG also prevented AMPK activation (Figure S8). The addition of DMKG was sufficient to mimic increased apoptotic cell death, and the Seahorse^®^ analysis indicated an increase in mitochondrial activity and in the glycolytic rate. Metformin abrogated all effects (Figure S8). Conditions of in vitro modelling and ex vivo validation did not show DMKG-induced oxidative damage (Figure S6f). However, the data support that alterations in energy and 1-C metabolism modifies the hepatic AMPK/mTOR signalling pathways responsible for the harmonization of apoptosis and autophagy as shown in clinical samples.

### 2.6. Analysis of Hepatic Transcriptome Signatures and Differentially Methylated Genomic Regions

**Figure 6:**
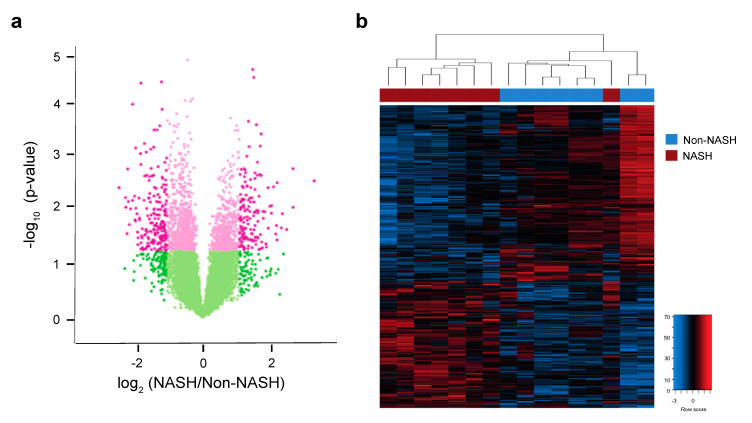
Hepatic transcriptome signature associated with NASH. (**a**) Volcano plot showing changes in mRNA expression in patients with or without NASH. Pink colouring indicates *p*-values < 0.05, while darker shading indicates absolute value of log2 (fold-change) in expression greater than 1. (**b**) Heatmap showing significantly differentially expressed genes with *p* < 0.05 and absolute value of log2 (fold-change) > 1. Unsupervised hierarchical clustering revealed a clear separation between NASH and non-NASH (shown as red and blue bars across the top of the heatmap, respectively). The colour range indicates low to high gene expression (blue to red, respectively).

We explored the existence of distinct hepatic transcriptome signatures and their association with methylated genomic regions. The metabolic differences in the livers of patients with NASH appeared to be driven by changes in gene expression profiles that enhanced glutamine uptake. The effects on α-KG, SAM, and SAH, essential metabolites in methyltransferase reactions, suggested epigenetic influences. To avoid the effect of metabolic confounders, we selected the livers of eight patients with and eight without NASH, all without metabolic comorbidities and matched for age, sex, and BMI. Unfortunately, RNA from paired biopsies did not achieve analytical quality in some and prevented assessment after surgery. Microarray analysis of mRNA transcripts identified a hepatic transcriptome signature composed of 345 genes, including 201 downregulated and 144 upregulated genes, that distinguished patients with and without NASH (Figure 6a,b, Tables S4 and S5). KEGG pathway analysis indicated that most of the genes with significantly lower expression in patients with NASH were involved in the biosynthesis and detoxification of metabolites, integration of insulin signalling with mitochondrial function, transport of metabolites that govern mitochondrial-cytosol crosstalk, and reversible transamination between alanine and α-KG to generate pyruvate and glutamate. Upregulated genes included those associated with cell adhesion and migration, extracellular matrix molecular remodelling, inflammation signalling and lipoprotein activity (Figure S9). The DNA methyltransferases catalyse the transfer of a methyl group from SAM to DNA; ten–eleven translocation (TET) enzymes oxidise 5-methylcytosines (5-mC) promote locus-specific reversal of DNA methylation and can be stimulated or inhibited in the presence of mitochondrial metabolites (Figure S10a). The relative abundance of metabolites in the livers of patients with and without NASH strongly suggests crosstalk between cellular energetics and DNA methylation (Figure S10b).

**Figure 7:**
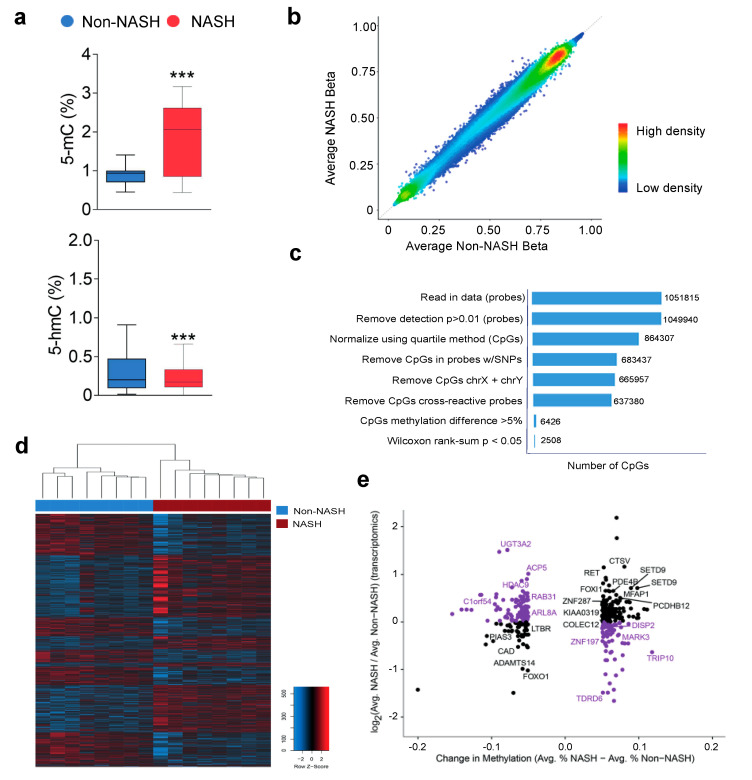
In liver DNA, differentially methylated genomic regions were associated with hepatic gene expression: (**a**) NASH affected the 5-methylcytosine-to-5-hydroxymethylcytosine conversion in genomic DNA with (**b**) stable bimodal distribution of CpG methylation; (**c**) there were 2508 differentially methylated CpGs between groups; (**d**) unsupervised hierarchical clustering identified a subset of 367 differentially methylated CpGs in promoters that distinguished the livers of patients with and without NASH. (**e**) The scatter plot shows changes in methylation and gene expression. Purple colouring indicates CpGs in promoters of genes whose expression goes up or down with promoter hypo- or hypermethylation, respectively and labels indicate significant correlations between methylation and gene expression. *** *p* < 0.001.

Measured DNA concentrations of 5-mC were significantly higher in patients with NASH than in patients without NASH (Figure 7a), and genome-wide hepatic DNA methylation screening resulted in largely stable cytosine 5 prime to guanine (CpG) methylation across the livers of patients with and without NASH (Figure 7b). Statistical analysis identified 2508 differentially methylated CpG sites that distinguished patients with and without NASH (Figure 7c,d). We focused on the differentially methylated CpG sites located in promoter regions that correlated with the mRNA microarray data, and we found biologically significant changes associated with 222 hypomethylated and 145 hypermethylated CpG sites (Table S6, Figure S11a and Figure 7e). Correlations between gene expression and candidate epigenetic modifiers were widely distributed across the genome, and KEGG pathway analysis revealed associations with hepatic reductase expression, mRNA surveillance and Coenzyme A, biliary acid and glycosphingolipid biosynthesis (Figure S11b,c). Our results suggest that DNA methylation is likely a mechanism by which transcription of key enzymes is regulated in NASH livers but the integrative analysis did not support a direct link with α-KG abundance. However, the significantly differential methylation in promoters of GAPDH, IDH3B, LDHAL6A, and NDUFB10 are highly suggestive. The use of more stringent criteria reduced the number of candidates to 11 CpGs, located in 11 loci, that exhibited significant changes in methylation in promoters that undergo decreases or increases in gene expression, respectively (Figure S12). These candidates retained the ability to distinguish between patients with and without NASH and highlight major effects in metabolic processes. The expression of significantly hypermethylated (DISP2, MARK3, TDRD6, TRIP10, and ZNF197) or hypomethylated (ACP5, ARL8A, C1orf54, HDAC9, RAB31, and UGT3A2) sets of genes in the livers of patients with NASH was examined individually via real-time quantitative PCR (Figure S11d). Significant transcriptional changes after NASH remission in a majority of candidates provide validation of reversible liver remodelling and highlight the importance of considering jointly hepatic transcriptomic and epigenetic markers in the assessment of NASH-associated adaptive responses.

## 3. Discussion

Experimental studies suggest that multiple and sequential hepatic insults may explain the progressive nature of the liver disease [14,15]. We recently reported that mitochondrial stress signals, adaptive inflammatory responses, and modulation of ROS generation in humans may act as potential initiators of liver disease and, consequently, therapeutic targets [3,6]. A previous study by our research group in a large series of patients with morbid obesity and various degrees of liver disease showed that LSG improves their metabolic disorders, including diabetes and hyperlipidaemia, and also liver disorders, especially steatosis and fibrosis. These beneficial effects of LSG were associated with a decrease in hepatic and circulating markers of oxidative stress and inflammatory cytokines. The results of this study suggested a sequential involvement of multiple cellular responses in the hepatic therapeutic effects of LSG [16]. The present study addresses this point through an exhaustive analysis at the histological, biochemical, metabolomic and epigenetic levels in a homogeneous group of patients with NASH. It is difficult to extrapolate conclusions to the general population, but the reversibility of NASH in patients with severe obesity undergoing surgery may represent a model to explore this topic.

Liver disease is significantly improved after surgery [16], and NASH remission reversed the oxidative stress observed in the livers of patients with NASH, restored mitochondrial dynamics, and resolved respiratory chain defects. NASH remission also abrogated the dysfunctional activation of mitochondrial stress-related adaptive functions via mitogen-activated protein kinase pathways. Interestingly, these alterations also distinguished the livers of patients with and without NASH reinforcing the concept of potential NASH predisposing factors. However, patients remain obese. Circulating adipokines and hepatokines are normalised after NASH remission and increased or decreased levels in patients with and without NASH potentiates crosstalk between adipose tissue and liver as an emerging paradigm that includes the gut, linking microbiota to the severity of liver disease [17,18].

The response of other cytokines also suggests major effects in liver metabolism and hepatocyte survival indicating that cell death may govern outcomes in NASH [19]. Indeed, apoptotic nuclei were significantly increased in NASH livers. We also found that increased apoptosis and decreased autophagy via mTORC1 hyperactivation were differential characteristics in the livers of patients with NASH. NASH remission inhibited mTORC1, reactivated autophagy and reversed apoptosis.

We have previously found that circulating α-KG may predict liver disease in obesity [20]. Hepatic metabolomics revealed a metabolic rewiring in which glutamine is reversibly favoured as an energy source in the livers of patients with NASH with perturbations in the methionine cycle, glutaminolysis and α-KG to succinate conversion. The hepatic mRNA expression of enzymes that regulate mitochondrial metabolism confirmed the metabolic effects and distinguished the livers of patients with and without NASH. In animal models, the overexpression of glutaminase 1 isoform (GLS1) and/or the underexpression of α-KG dehydrogenase (α-KGDH) activated mTORC1 and emphasised the importance of glutaminolysis and α-KG in regulating the dynamic adaptability of the liver [21,22]. Indeed, intracellular α-KG and mTORC1 activation play a major role in the metabolic pathways that lead to self-renewal and differentiation [23]. In humans, the most important clinical factor to assess the progressive fate of liver disease towards hepatocellular carcinoma in obesity is the long duration of NASH [24]. Hence, NASH remission is a primary therapeutic goal.

There is no licensed drug to treat NASH. However, there is growing evidence that mTORC1 activation and/or AMPK activation reverse glutaminolysis-mediated apoptosis and might improve liver damage [25]. We here demonstrate in in vitro and ex vivo models that increased intracellular α-KG levels are sufficient to recapitulate in hepatocytes metabolomic findings in NASH, including alterations in mitochondrial activity and glycolytic rate. Critically, metformin reverses the metabolic rewiring. The possibility to extend the use of this old drug in patients with chronic liver disease is a possibility deserving further research [26].

Changes in metabolites from energy and 1-C metabolism prompted us to explore the role of transcriptomic and epigenetic adaptive responses in NASH pathogenesis. We identified specific signatures that differentiated patients with and without NASH. The poor overlap between published signatures indicates the limitations imposed by differences in methods, design, and bioinformatics tools [27,28]. The integrative analysis of transcriptomic and epigenetic deregulation focused on differentially methylated promoter regions that correlated with gene expression identified a gene profile that distinguished patients with and without NASH. Integrative analysis indicated that DNA methylation is likely a mechanism by which transcription of key enzymes is regulated in NASH livers but did not support a direct link with α-KG abundance. However, the findings support the dissemination of mitochondrial initiators that mirror multifaceted defective responses, extracellular matrix molecular remodelling and inflammation signalling, with potential in future hepatic metabolic research.

In conclusion, NASH remission illustrates the need for multiple therapeutic targeting and the importance of mitochondrial dysfunction. The increased glutaminolysis-induced α-KG production may be a pathogenic candidate to control promotion of the NASH phenotype.

## 4. Materials and Methods

### 4.1. Participants

This post hoc study included new objectives derived from a previous prospective longitudinal study searching for plasma biomarkers of obesity-associated liver disease [6]. Among the patients undergoing bariatric surgery who donated intraoperative liver samples, we recruited participants on the opposite sides of the spectrum, namely patients with and without NASH (n = 31, each), who were matched for sex, age, body mass index (BMI), and incidence of metabolic comorbidities. As per protocol, metformin was discontinued before surgery. The patients with NASH provided a second biopsy one year after the procedure by percutaneous needle puncture. The quality of the liver samples was assessed as described [29], and the nonalcoholic fatty liver disease activity score (NAS) [30] was used to normalise the histological features. Details may be found in the Supplementary Materials.

### 4.2. Analytical Methods

Hepatocyte mitochondria were examined by transmission electron microscopy as reported [31]. Portions of liver tissue were used for immunoblotting, immunocytochemical [32], metabolomic [7,33], transcriptomic, and epigenetic analyses. The data from gene expression microarrays and the assessment of differentially methylated genes required specific bioinformatics tools in R [34,35,36]. Cultured HepG2 cells and flow cytometry analyses were employed to confirm the effect of α-KG on mitochondrial metabolism and apoptosis. For multianalyte cytokine detection, we used a customised bead-based Luminex^®^ assay (R&D systems, Minneapolis, MN, USA), and blood samples from lean controls without liver disease were used for comparisons (n = 31). A detailed description of the methods used is shown in the Supplementary Materials (Supplementary Materials, Figure S11 and Tables S6 and S7).

### 4.3. Statistical Analysis

The Mann–Whitney or Wilcoxon test (nonparametric) was used to establish differences between groups. Differences were considered statistically significant when *p* < 0.05. The chi-square test was used to compare categorical variables. The results are shown as the mean ± SEM unless otherwise stated. The Benjamini–Hochberg procedure was used for controlling false discovery rates in multiple comparisons. We used the SPSS 22·0 package (IBM Corp. Armonk, NY, USA) and R version 3·4. Qualitative and quantitative analysis B·06·00 software (Agilent Technologies, Santa Clara, CA, USA) was used to detect and quantify the amounts of metabolites, and MetaboAnalyst 4·0 was used to generate the scores/loading plots, heatmaps, and multivariate and random forest analyses.

### 4.4. Ethics

This study was approved by the Comitè d’Ètica i Investigació en Medicaments (Institutional Review Committee) of the Institut d’Investigació Sanitària Pere Virgili (OM-NAFLD, ESO3/18012013, INFLAMET/15-04/4proj7 and OBESPAD/14·07-31proj3) and fully informed written consents were obtained from the participants.

## Data Availability

The raw metabolomics, transcriptomics, and epigenetics data were deposited at the *Universitat Rovira i Virgili* repository and will be accessible upon publication of the manuscript.

## Supplementary Materials

The following supporting information can be downloaded at: https://www.mdpi.com/article/10.3390/ijms23147830/s1. References are cited [37,38,39,40,41].
