# Supplementary material for: Laparoscopic Sleeve Gastrectomy in Patients with Severe Obesity Restores Adaptive Responses Leading to Nonalcoholic Steatohepatitis
Source: Int J Mol Sci. 2022 Jul 15;23(14):7830. doi: 10.3390/ijms23147830 (PMC9320342; doi:10.3390/ijms23147830)
Supplement: Supplementary file 1 [file ijms-23-07830-s001.zip › ijms-1807556-supplementary.pdf]

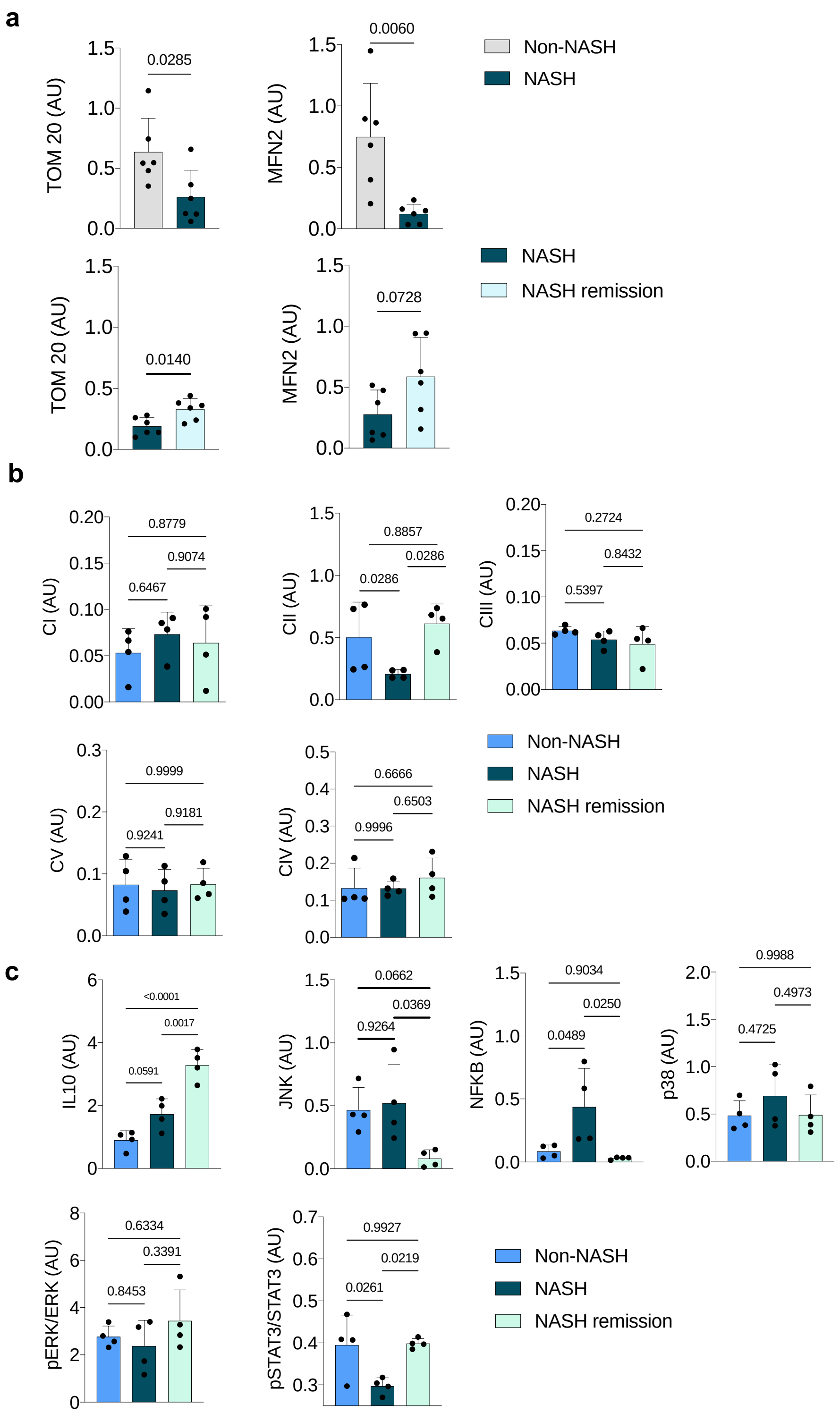

Supplementary Figure S1. NASH remission is associated with reduced liver mitochondrial damage, oxidative stress and inflammation. Western blot quantification for selected markers in the livers of patients with and without NASH and patients with NASH before and after surgery. P-values were calculated by the Mann-Whitney *U* test. Results are shown as means and SD.

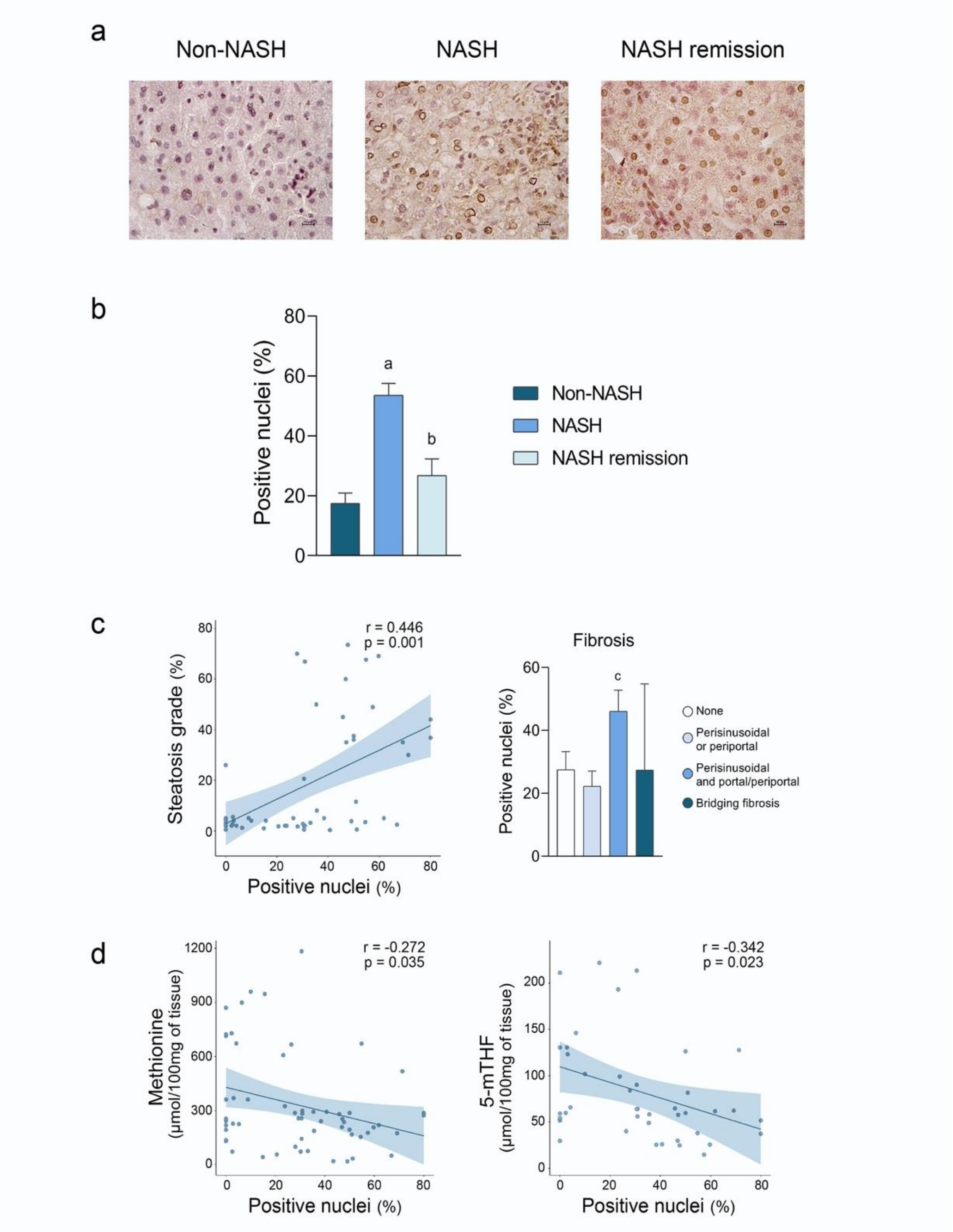

Supplementary Figure S2. Terminal deoxynucleotidyl transferase-mediated dUTP nick end labelling (TUNEL) for the measurement of apoptotic nuclei. (a) Representative micrographs corresponding to liver biopsies from a patient without NASH and 3.1% positive nuclei, a patient with NASH and 71·3% positive nuclei, and a post-surgery biopsy with 25·3% positive nuclei. Nuclei with positive staining appear brown. (b) Patients with NASH showed a significant increase in the proportion of nuclei positive for TUNEL compared to patients without NASH. These values decreased after surgery. (c) The proportion of TUNEL positive nuclei significantly correlated with the steatosis grade and was higher in patients with perisinusoidal and portal/periportal fibrosis. (d) The proportion of TUNEL positive nuclei showed inverse significant correlations with hepatic methionine and 5-mehtyl tetrahydrofolate (5-mTHF) concentrations. ^{a} at least $p<0{\cdot}05$ with respect to patients without NASH; ^{b} at least $p<0{\cdot}05$ with respect to patients with NASH; ^{c} at least $p<0{\cdot}05$ with respect to patients without fibrosis or perisinusoidal or periportal fibrosis. P-values were calculated by the Mann-Whitney *U* test. Results are shown as means and SD. Correlations were calculated with the Spearman's rho test.

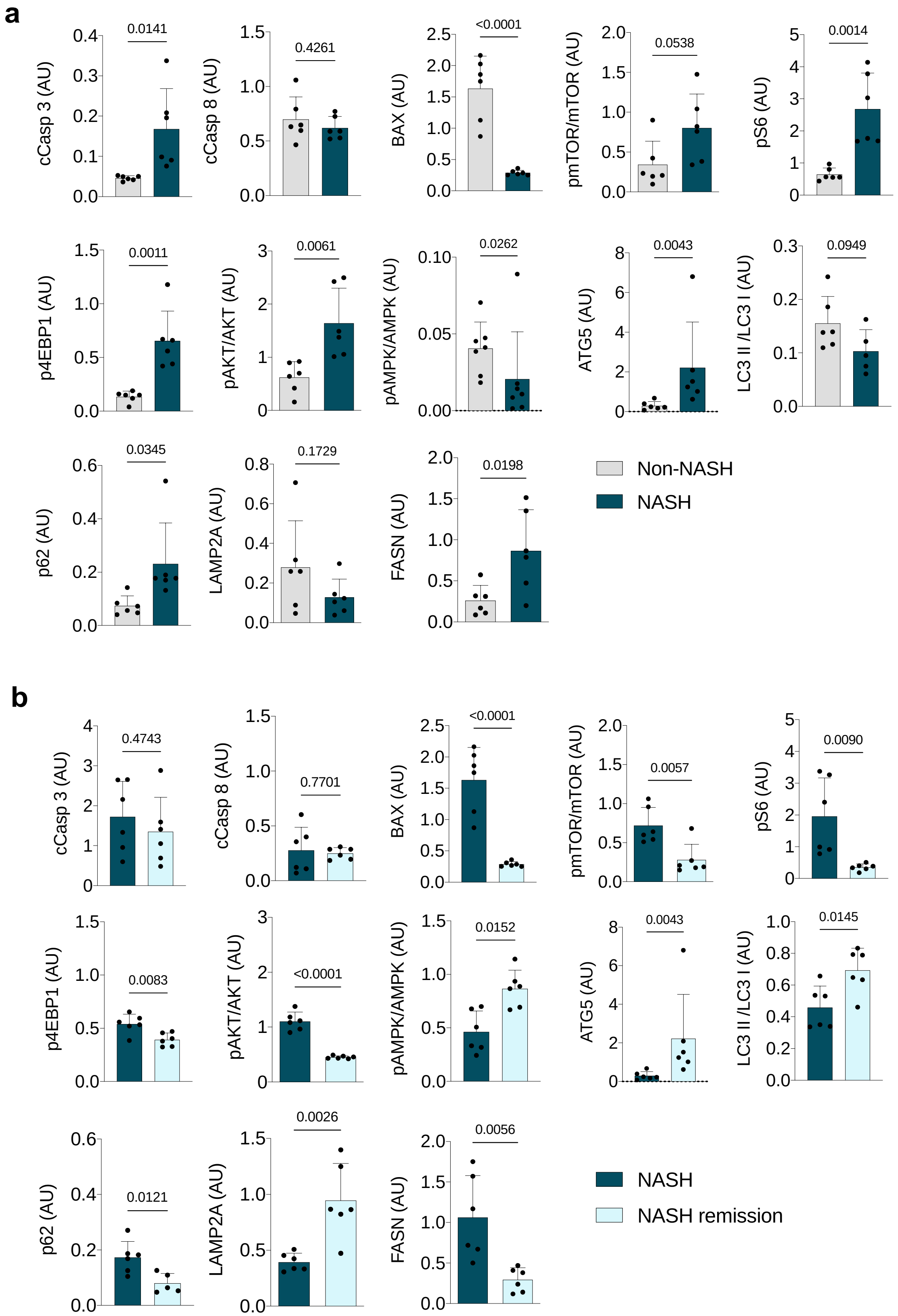

Supplementary Figure S3. **mTORC1 signalling and NASH remission.** Western blot quantification for selected markers in the livers of patients with and without NASH (a) and NASH patients before and after surgery (c). P-values were calculated by the Mann-Whitney *U* test. Results are shown as means and SD.

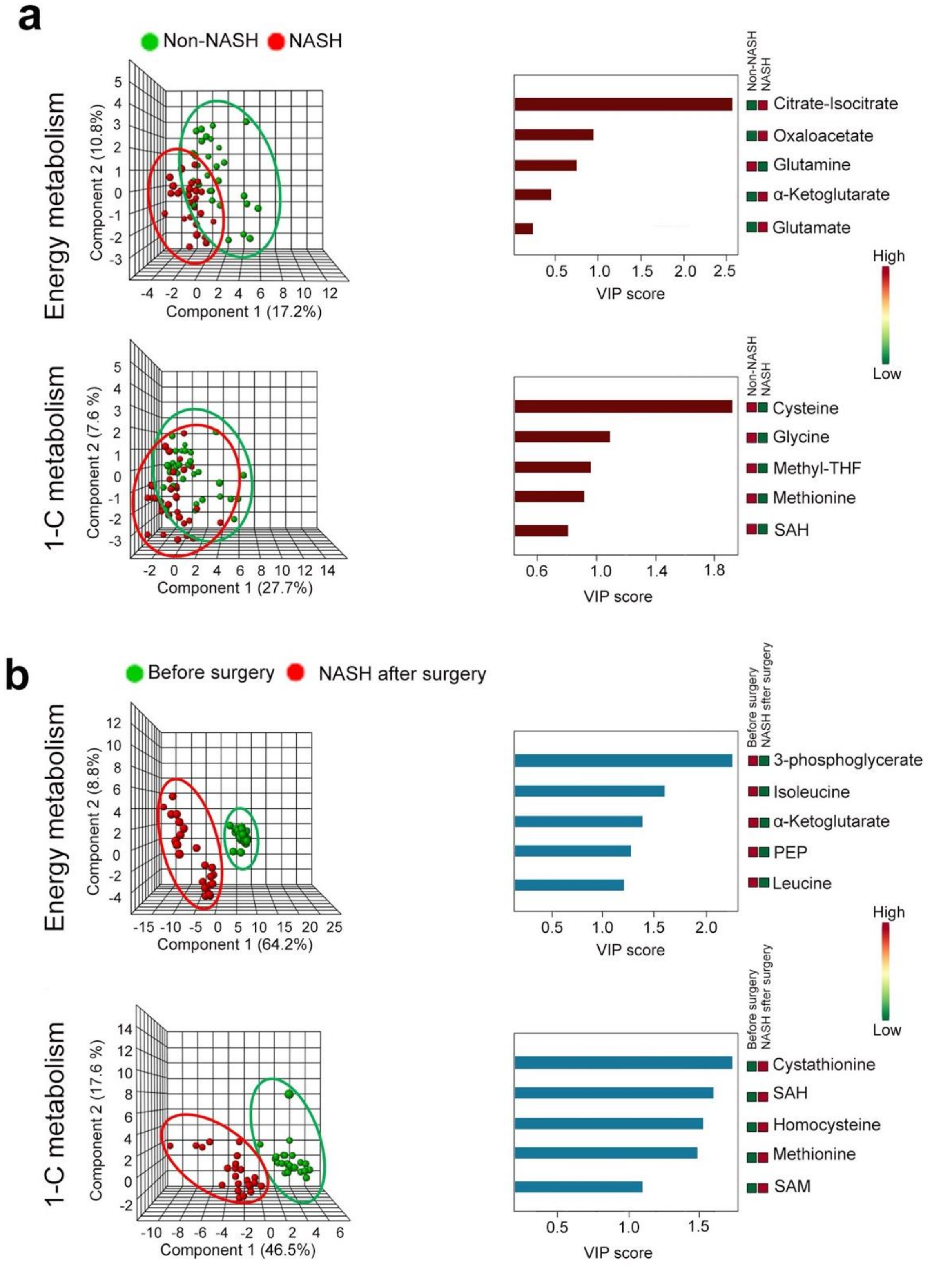

Supplementary Figure S4. Energy and one-carbon metabolism distinguished livers of patients with and without NASH: the effect of NASH remission. (a) Principal component analysis and variable importance scores in random forests indicated the relative importance of different metabolites in distinguishing the livers of patients with and without NASH. (b) The same analyses retained the importance of α-KG-related metabolites and metabolites from the methionine cycle in metabolic reversal after NASH remission.

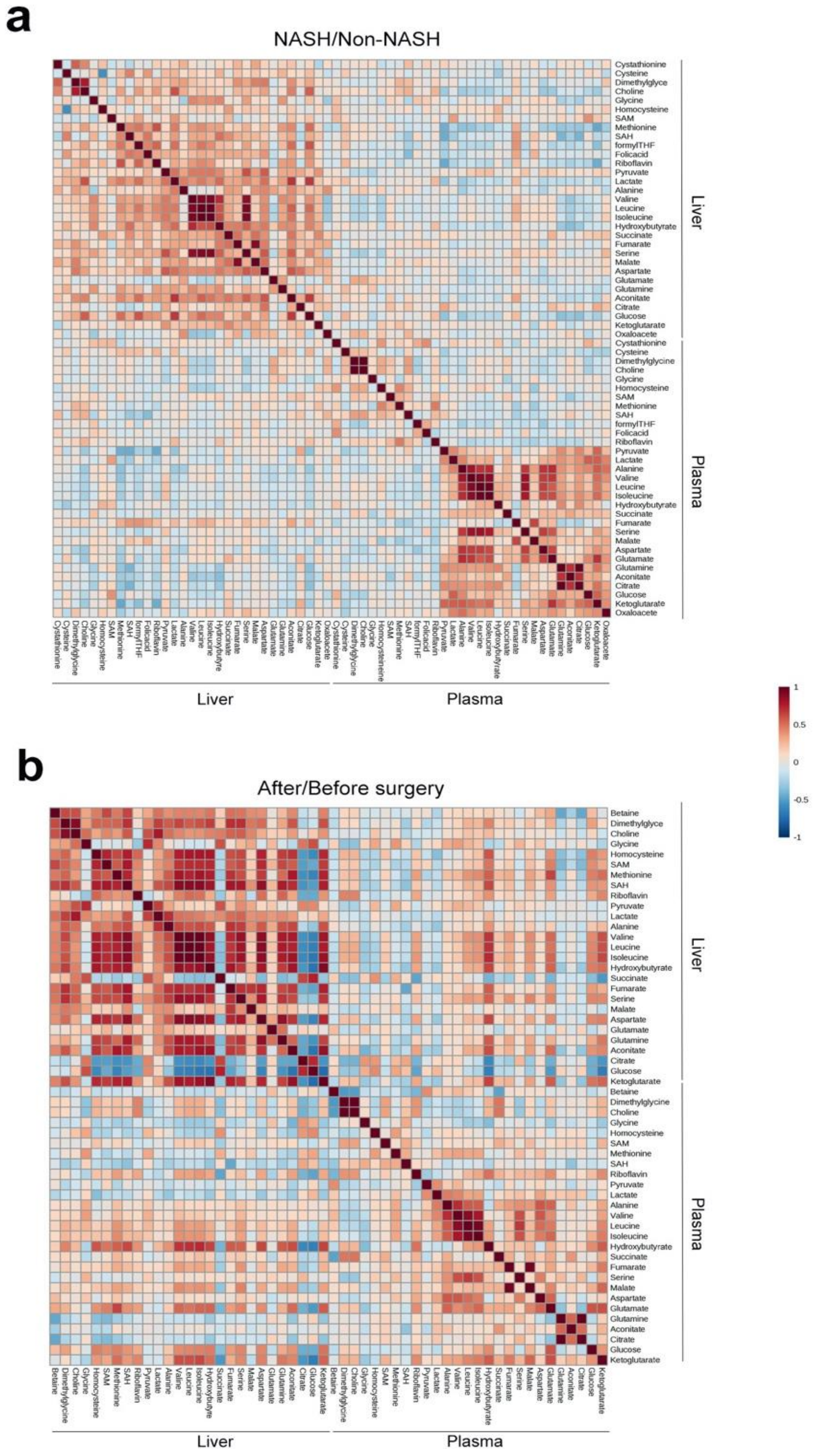

Supplementary Figure S5. The reliability of plasma measurements compared with the liver metabolome. Assessment of the reliability of linear regression estimates and exploratory factor analysis via a correlation matrix between the plasma and liver metabolites in (a) the livers of patients with and without NASH and (b) variations after NASH remission. Collectively, the data displayed a similar response of the metabolome in the plasma and liver. The effect of plasma fumarate on the levels of numerous liver metabolites appeared to be significant when comparing the livers of patients with and without NASH. Similarly, α-KG, glutamine and hydroxybutyrate in the plasma might be associated with the metabolic response in NASH remission. Spearman's correlation was used to decrease susceptibility to outliers.

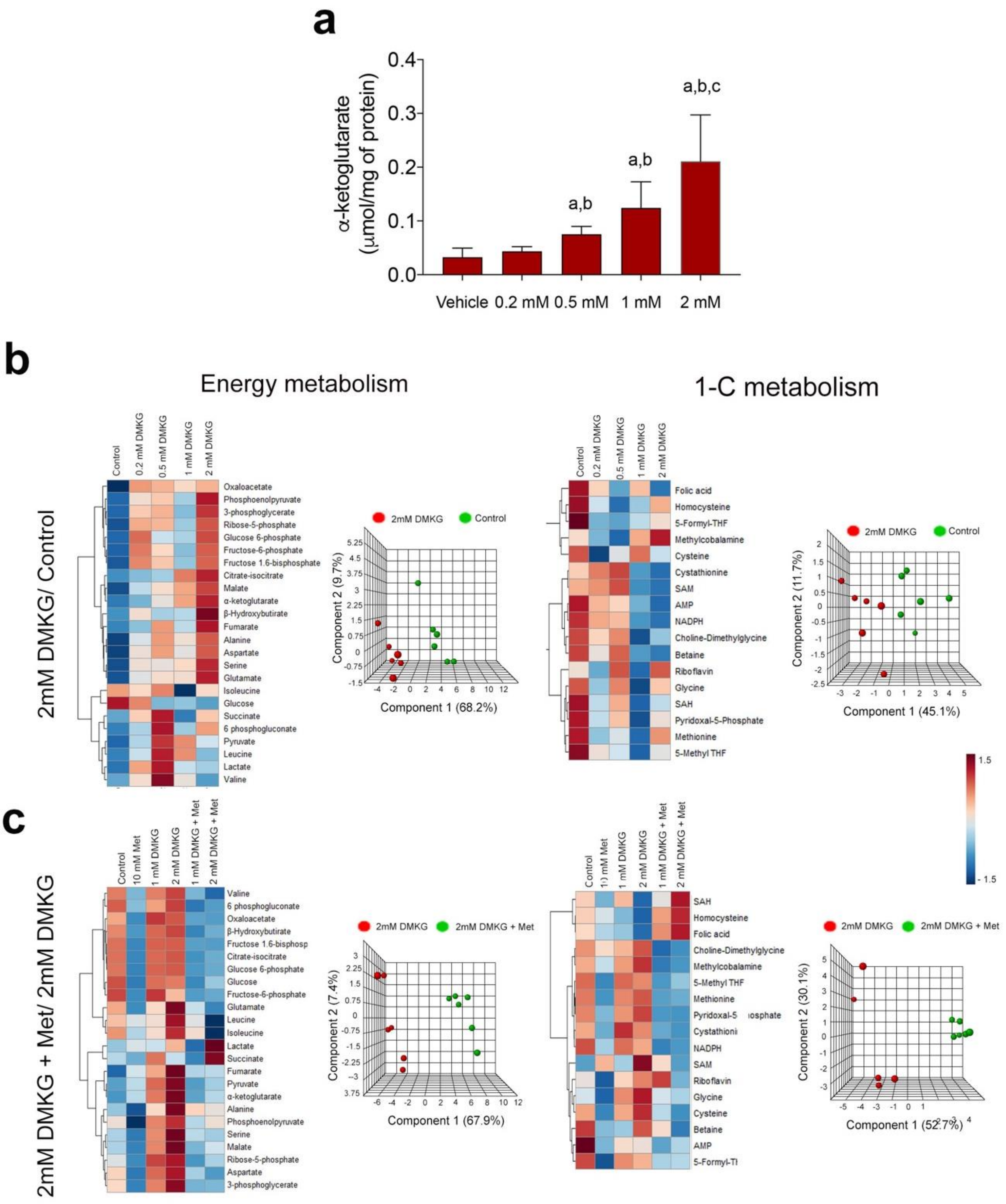

Supplementary Figure S6. Increased hepatocyte α-KG levels affect energy and one-carbon metabolism. (a) Increasing (from 0·2 mM to 2 mM) supplementation with a cell-permeable α-KG analog increased the intracellular α-KG levels. [a]at least p<0·05 compared to the controls, [b]at least p< 0·05 compared to 0·2 mM, [c]at least p<0·05 compared to 0·5 mM. (b) Heatmaps and principal component analysis indicated dose-dependent changes in metabolites that distinguished the treated and untreated cells. (c) The addition of metformin abrogated most metabolic changes induced by α-KG supplementation. P-values are calculated by the Mann-Whitney *U* test. Results are shown as means and SD.

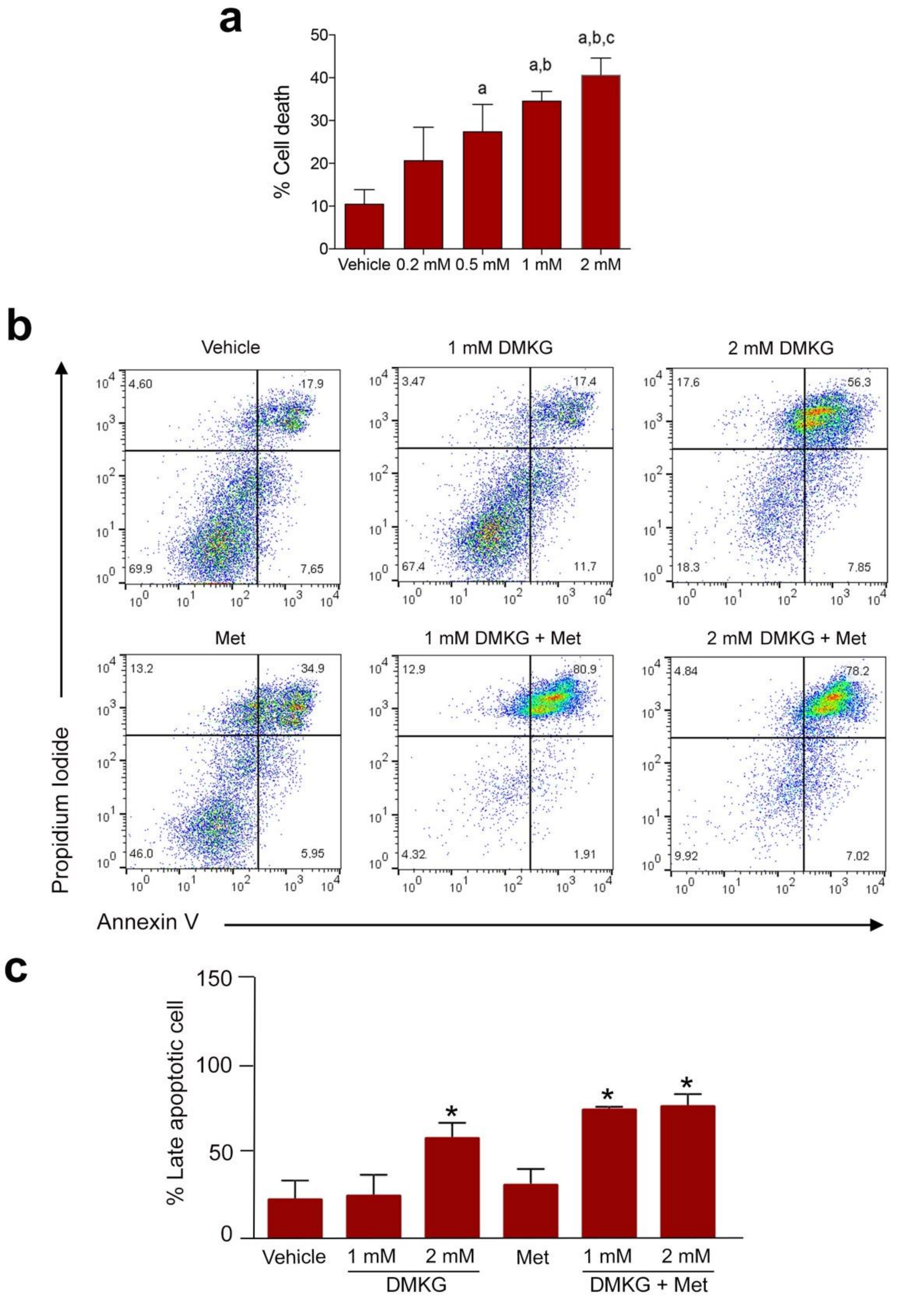

Supplementary Figure S7. Increased hepatocyte α-KG levels promote death in cultured hepatocytes. (a) Increasing (from 0·2 mM to 2 mM) supplementation with a cell-permeable α-KG analog increased cell death. [a]at least p<0·05 compared to controls, [b]at least p< 0·05 compared to 0·2 mM, [c]at least p<0·05 compared to 0·5 mM. (b) Flow cytometry analysis of the cells stained with annexin V and propidium iodide indicated an increase in apoptosis caused by high intracellular α-KG levels. (c) The number of late apoptotic cells confirmed that metformin may potentiate the effect of high α-KG levels. Results expressed as mean ± s.e.m. *p* values are determined by Wilcoxon rank-sum test (a) or *p<0·05 (ANOVA post hoc Bonferroni test in quintuplicate) in (c).

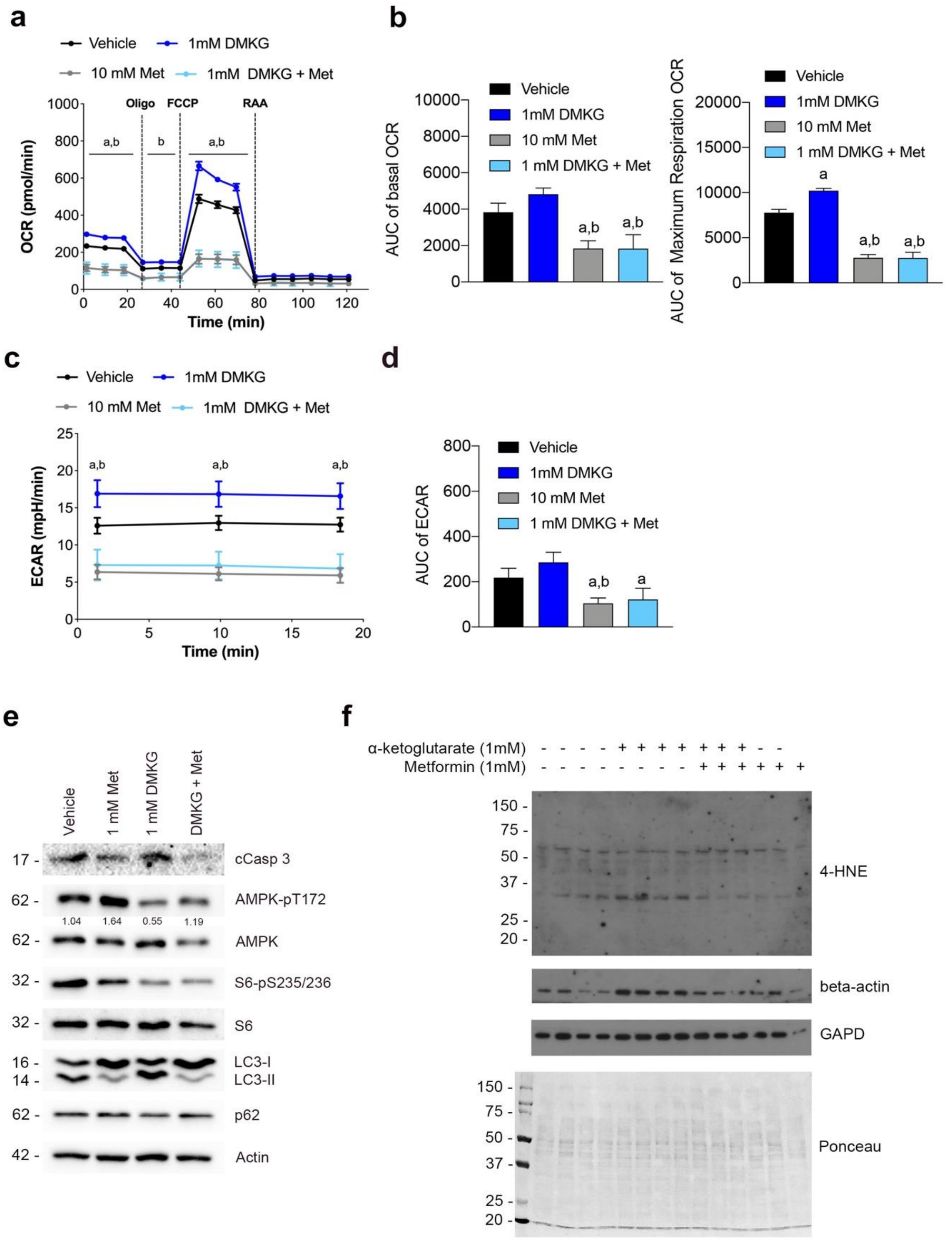

Supplementary Figure S8. Metformin regulates mitochondrial function in primary hepatocytes cells. (a,b). Oxygen consumption rate (OCR) and (c,d) extracellular acidification rate (ECAR) of primary hepatocytes cells revealed that supplementation with metformin abrogated α-KG-induced key cellular functions, as mitochondrial respiration and glycolysis. (e) Representative western blots of selected markers indicated that metformin also abolishes these effects by increasing AMPK phosphorylation. (f) Representative western blots of 4-hydroxynonenal (4-HNE) showed that the increase of intracellular α-KG did not alter the oxidative stress in the primary hepatocytes cells. [a] at least $p<0{\cdot}05$ compared to control, [b] at least $p< 0{\cdot}05$ compared to 1 mM DMG, [c] at least $p<0{\cdot}05$ compared to 10 mM metformin. Results expressed as mean ± s.e.m. *p* values are determined by One or two -way ANOVA with Tukey's post-hoc test.

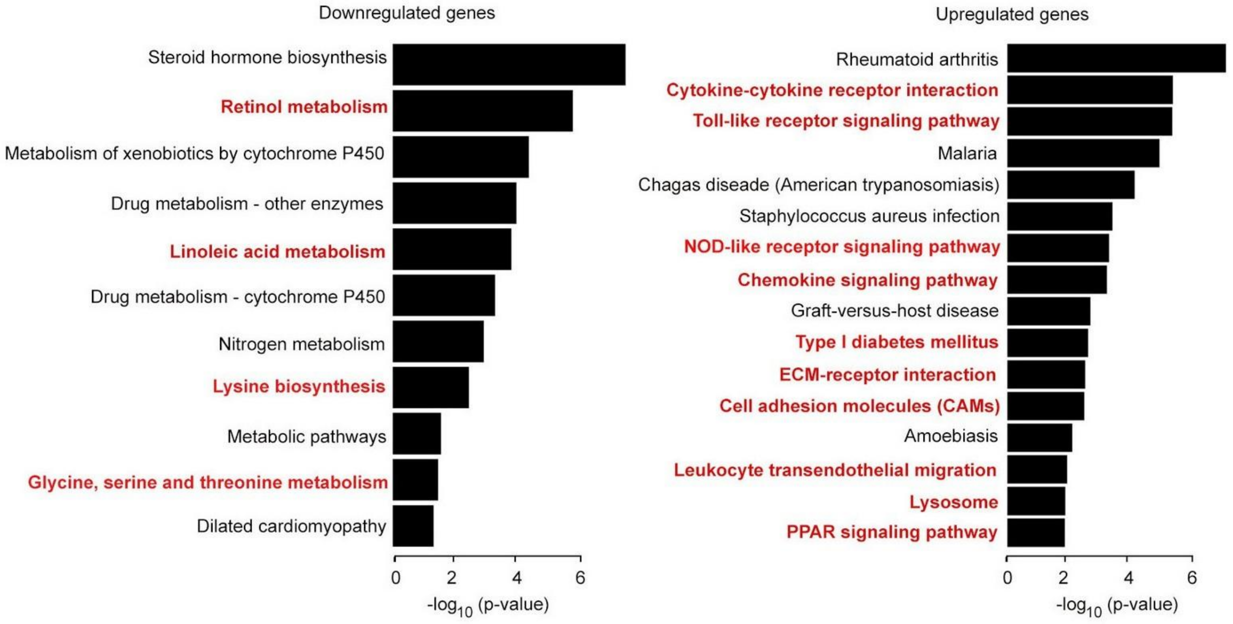

Supplementary Figure S9. KEGG pathway analysis of NASH and non-NASH phenotypes. The functional annotation of differentially expressed genes (upregulated and downregulated on the right and left, respectively) between patient cohorts indicated an association with biological processes involved in metabolism and disease.

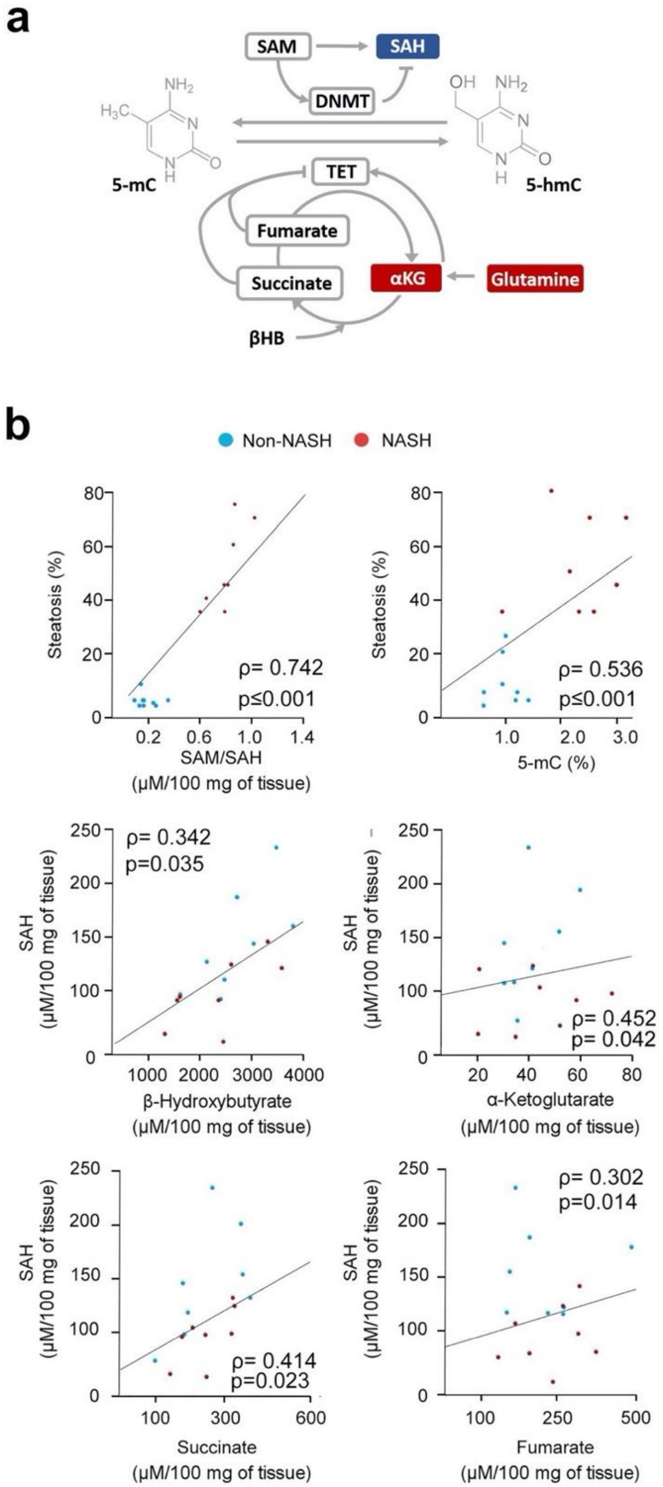

Supplementary Figure S10. Metabolites influence DNA methylation. (a) Several families of enzymes affect DNA methylation and require methyl donors and/or relative concentrations of mitochondrial metabolites. (b) Steatosis correlated with the amount of methyl donors and 5-methylcytosine. Hepatic mitochondrial metabolites are interrelated and distinguished the livers of patients with and without NASH. Correlations were calculated with the Spearman's rho test

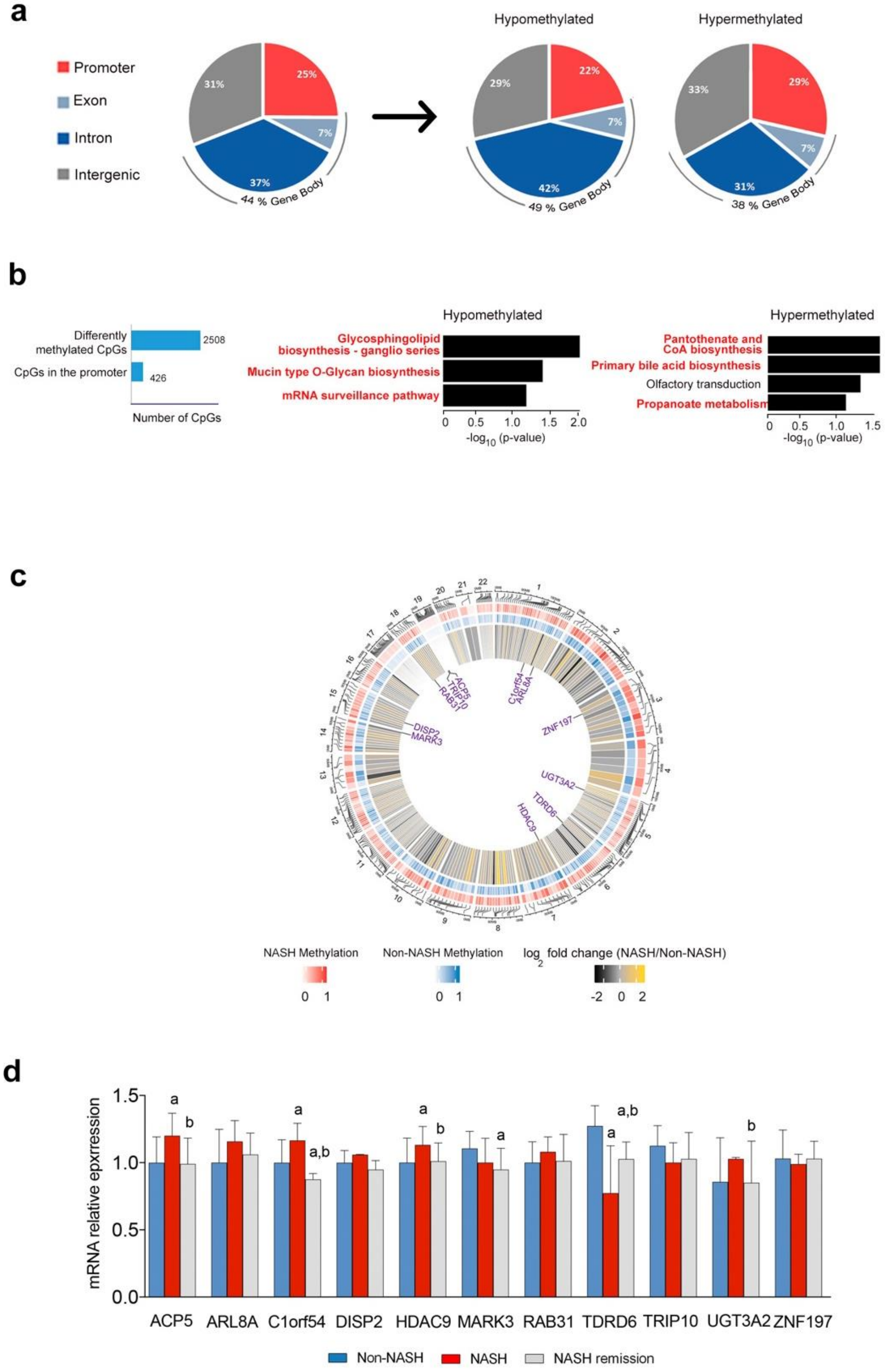

Supplementary Figure S11. Methylated CpG sites in livers of patients with NASH are not uniformly distributed in the genome. (a) Overall distribution of significantly differentially methylated CpG sites in promoter, exon, intron and intergenic regions indicated as percentages. (b) KEGG pathway analysis of gene expression in hypo- and hypermethylated promoter regions. Of the 2508 differentially methylated CpGs from the experiment, 426 are localised to promoters (we filtered for those annotated as TSS1500 or TSS200), as summarised in the blue bar chart on the left. (c) Circos plot showing chromosome locations of the subset of 367 CpGs for which we have mRNA microarray data (of the 426 from (b)) from Figures 7 and S8, with red and blue layers indicating localization and methylation levels in NASH and Non-NASH patients, respectively. The interior layer shows log2 (fold-change) in mRNA levels between NASH and Non-NASH patients, and text labels indicate CpGs highlighted in purple in Figure 7e. (d) Transcriptome response in selected genes using quantitative real-time PCR. [a] At least $p<0.05$ compared to non-NASH livers, [b] at least $p<0{\cdot}05$ compared to NASH livers. Results expressed as means and SD. *p* values are determined by Wilcoxon rank-sum test.

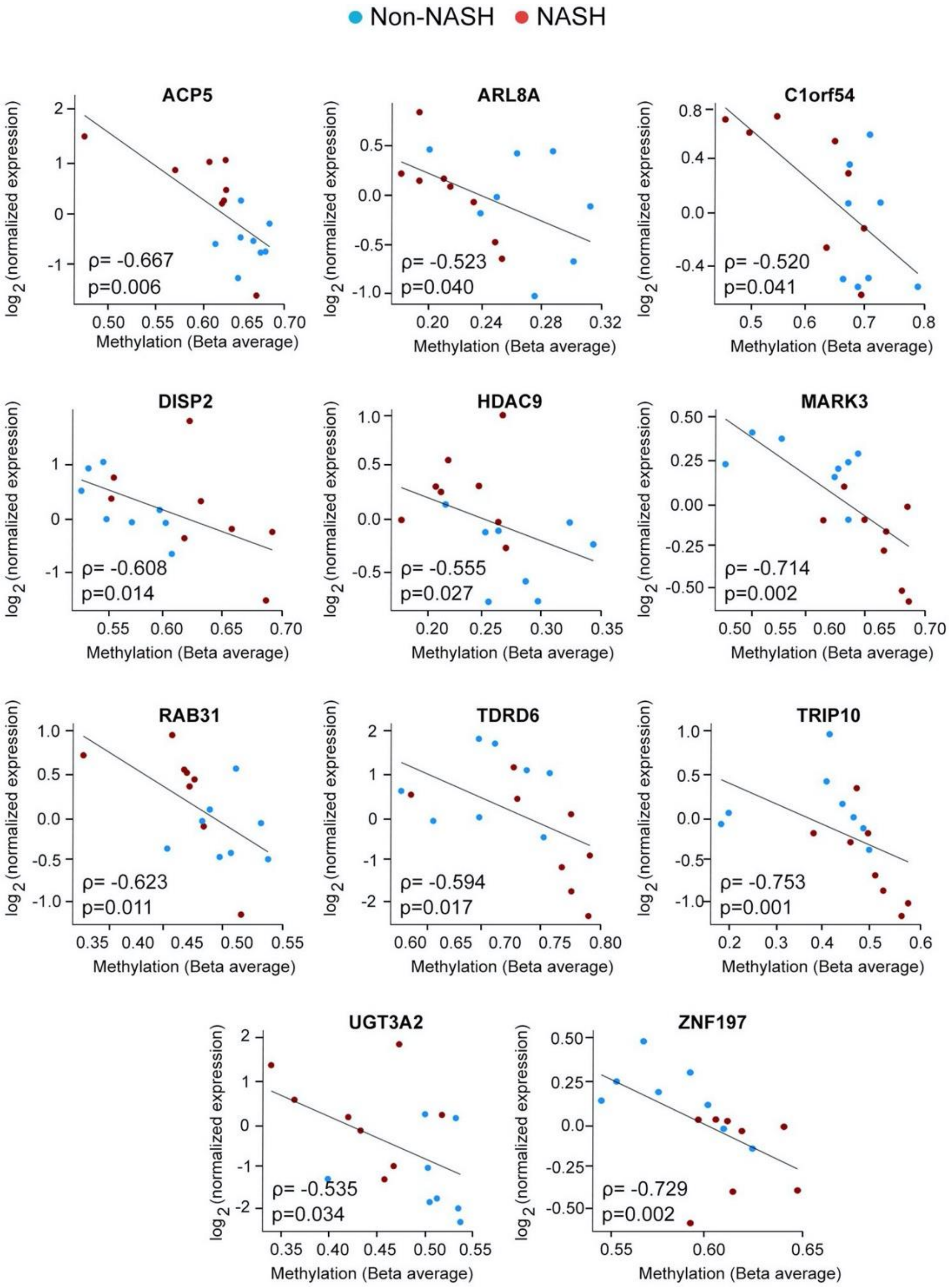

Supplementary Figure S12. Representative DNA methylation and mRNA expression measured in candidate CpG sites located in promoter regions. Spearman's correlation plots of DNA methylation (beta average) versus gene expression (log2 expression) in the livers of patients with and without NASH for the 11 candidates with inverse correlation between DNA methylation and mRNA expression. p-values determined by Spearman cor.test() in R. ACP5, acid phosphatase 5, tartrate-resistant; ARL8A, ADP ribosylation factor-like GTPase 8A; C1orf54, chromosome 1 open reading frame 54; DISP2, dispatched RND transporter family member 2; HDAC9, histone deacetylase 9; MARK3, microtubule affinity-regulating kinase 3; RAB31– RAB31, member RAS oncogene family; TDRD6, tudor domain-containing 6; TRIP10, thyroid hormone receptor interactor 10; UGT3A2, UDP glycosyltransferase family 3 member A2; ZNF197, zinc finger protein 197.

Supplementary Figure S13. Uncropped western blots corresponding to the main figures. M: Western blot protein marker. The numbers to the left of the blots are the molecular weights.

Figure 1b

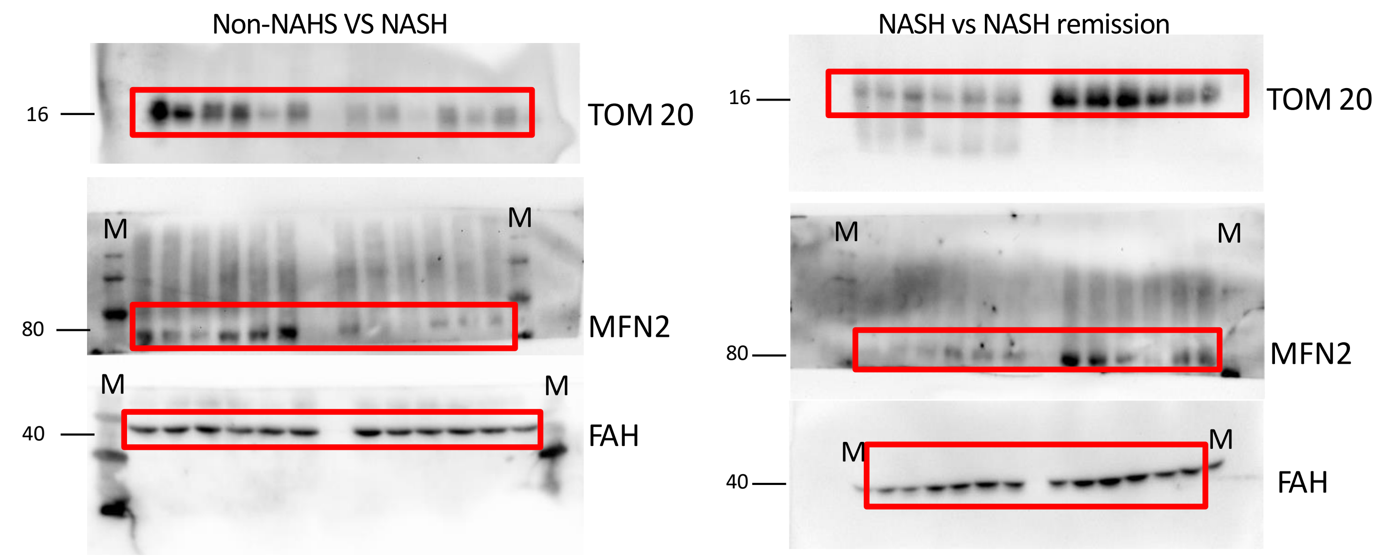

Figure 1c

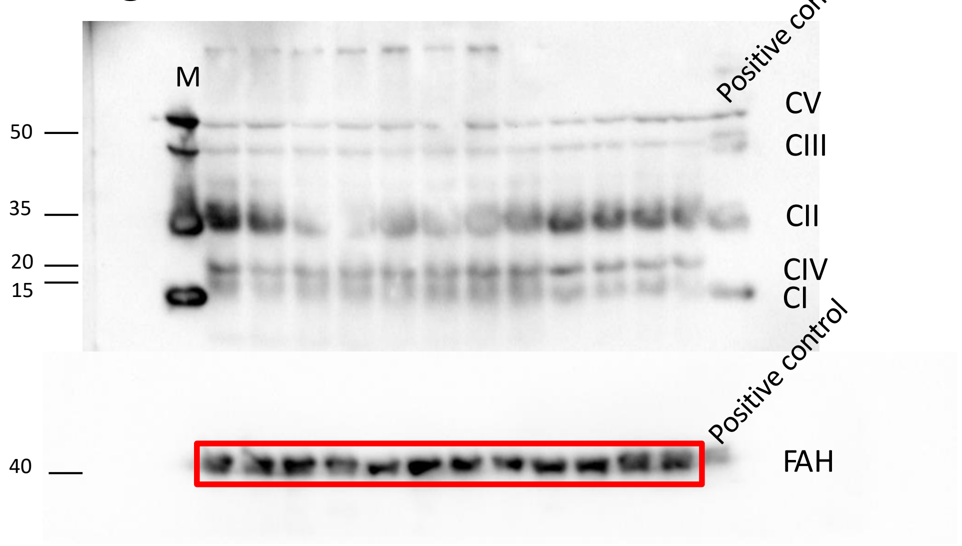

Figure 2b

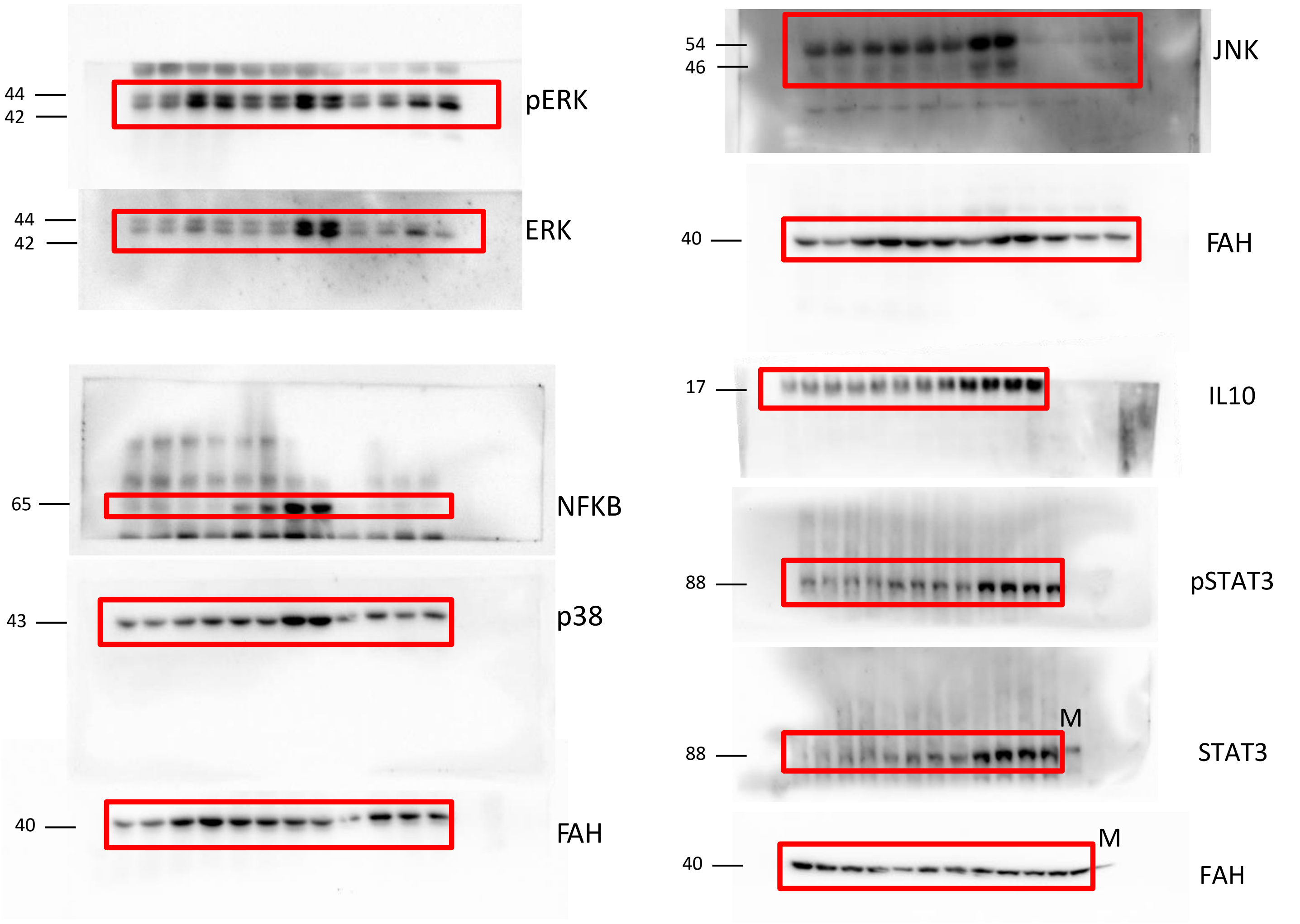
44
42
pERK
44
42
ERK
65
NFKB
43
p38
40
FAH
54
46
JNK
40
FAH
17
IL10
88
pSTAT3
M
88
STAT3
M
40
FAH

Figure 3B

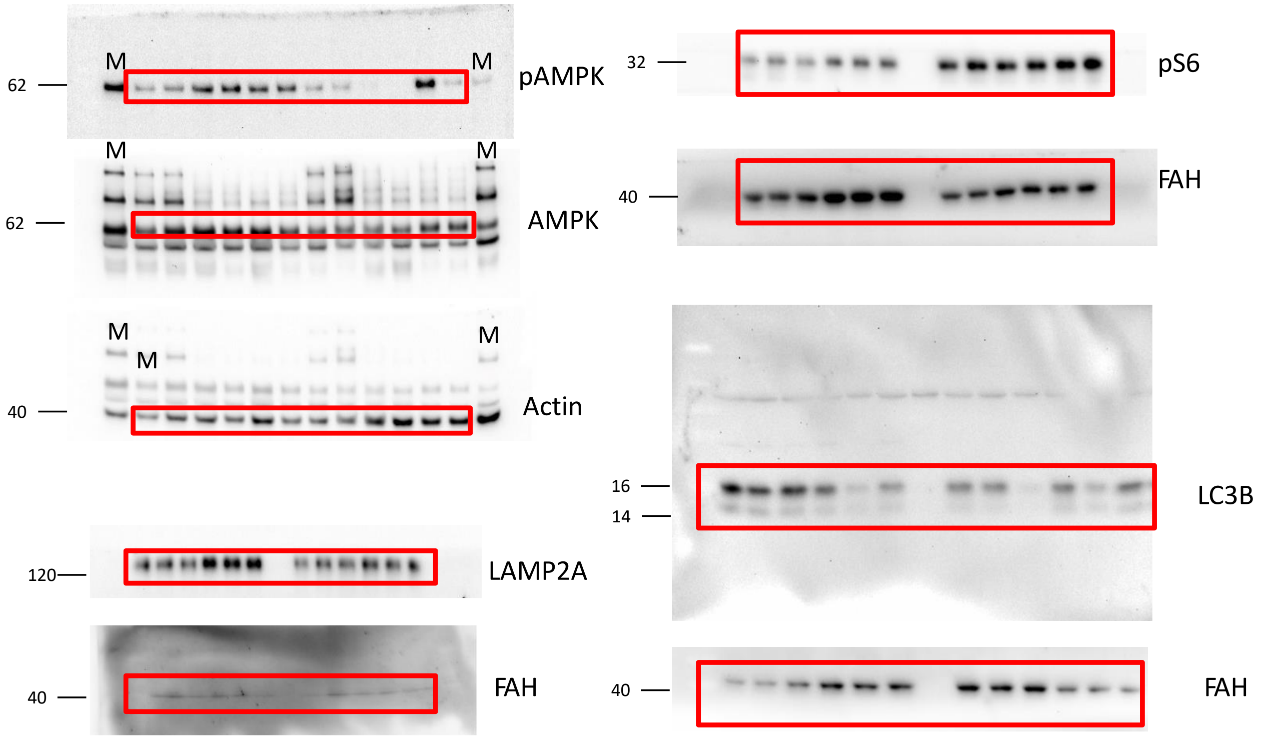

Figure 3B

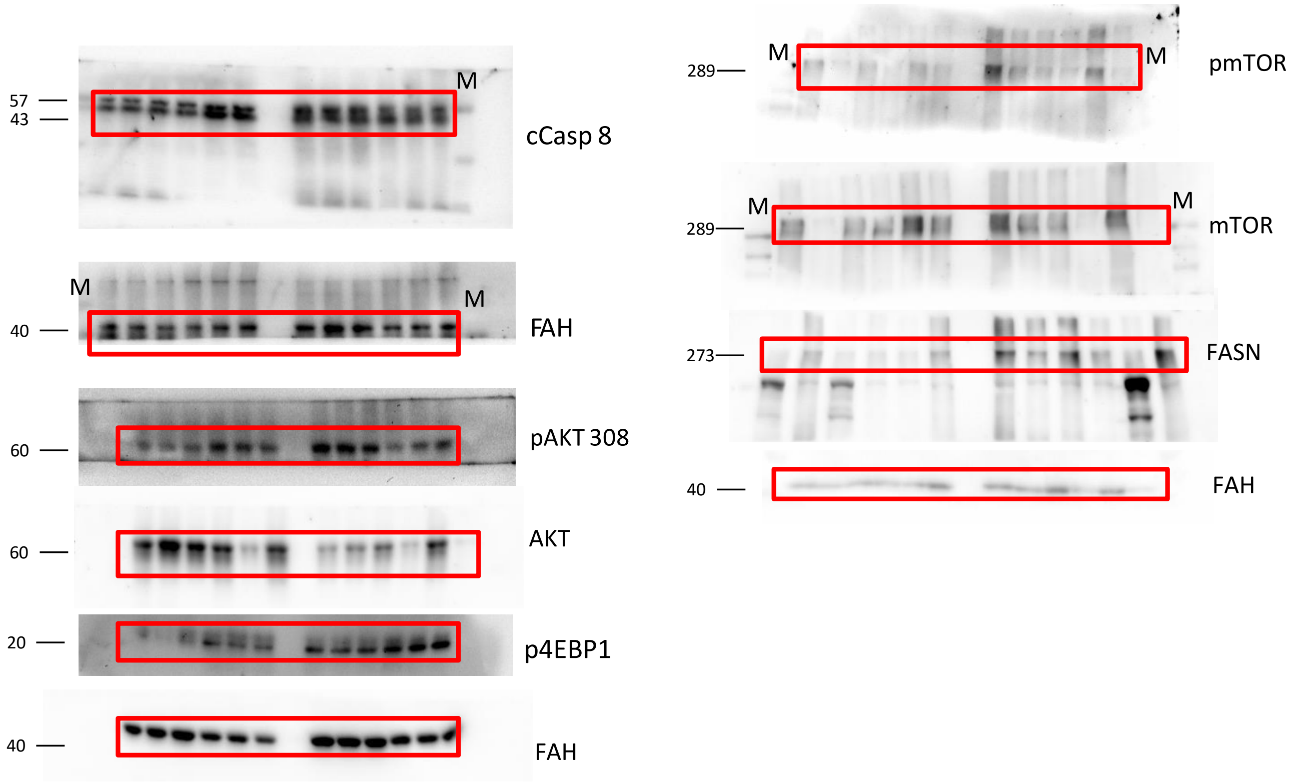

Figure 3B

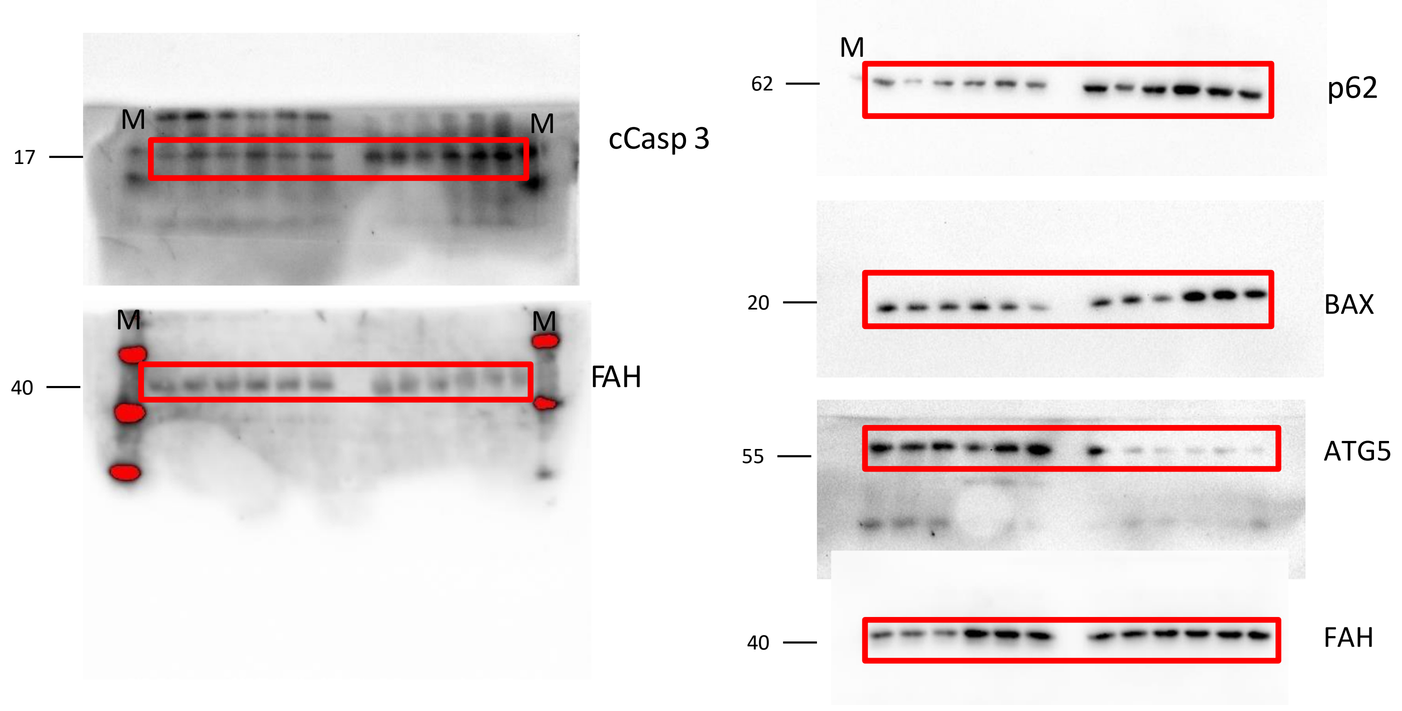

Figure 3D

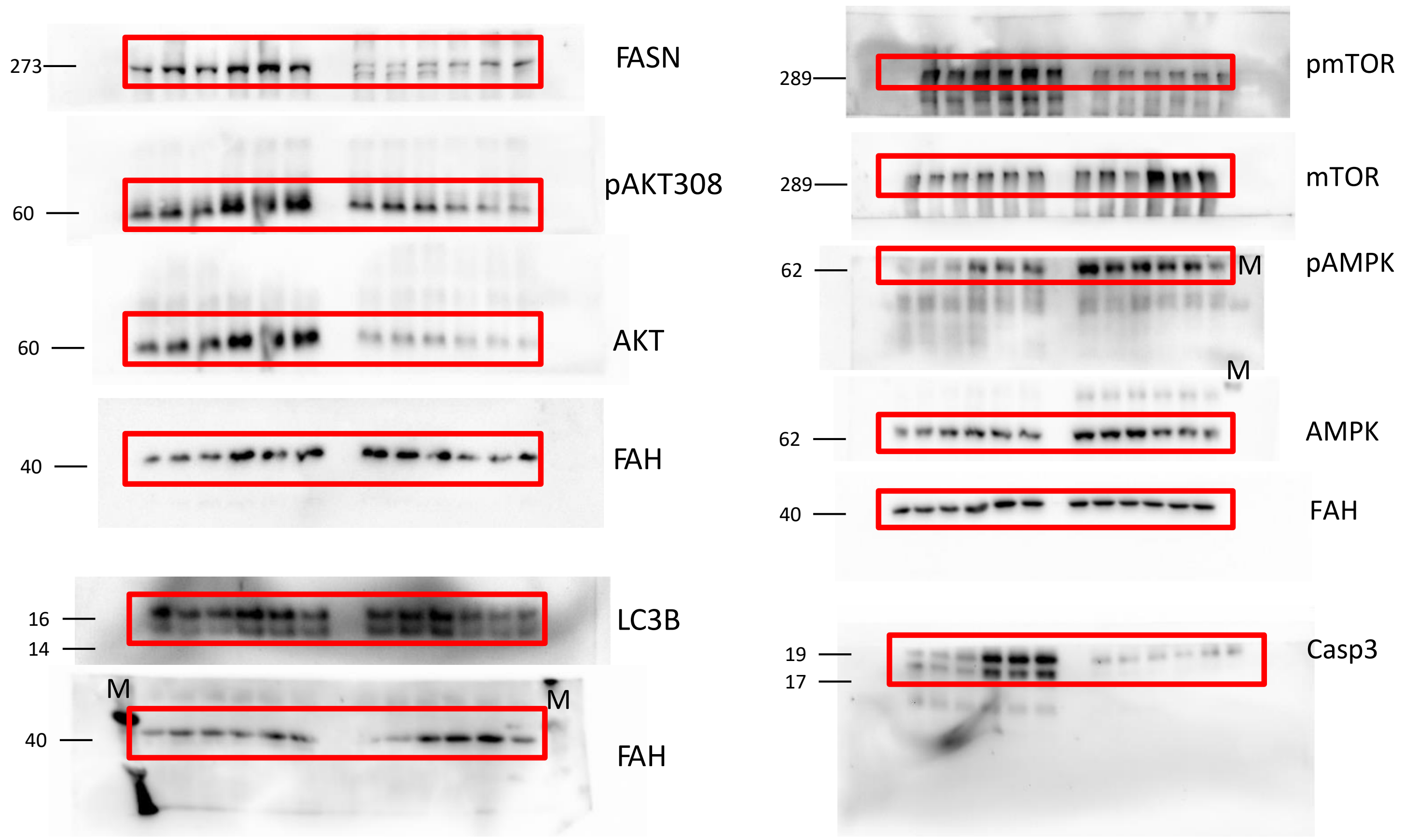

Figure 3D

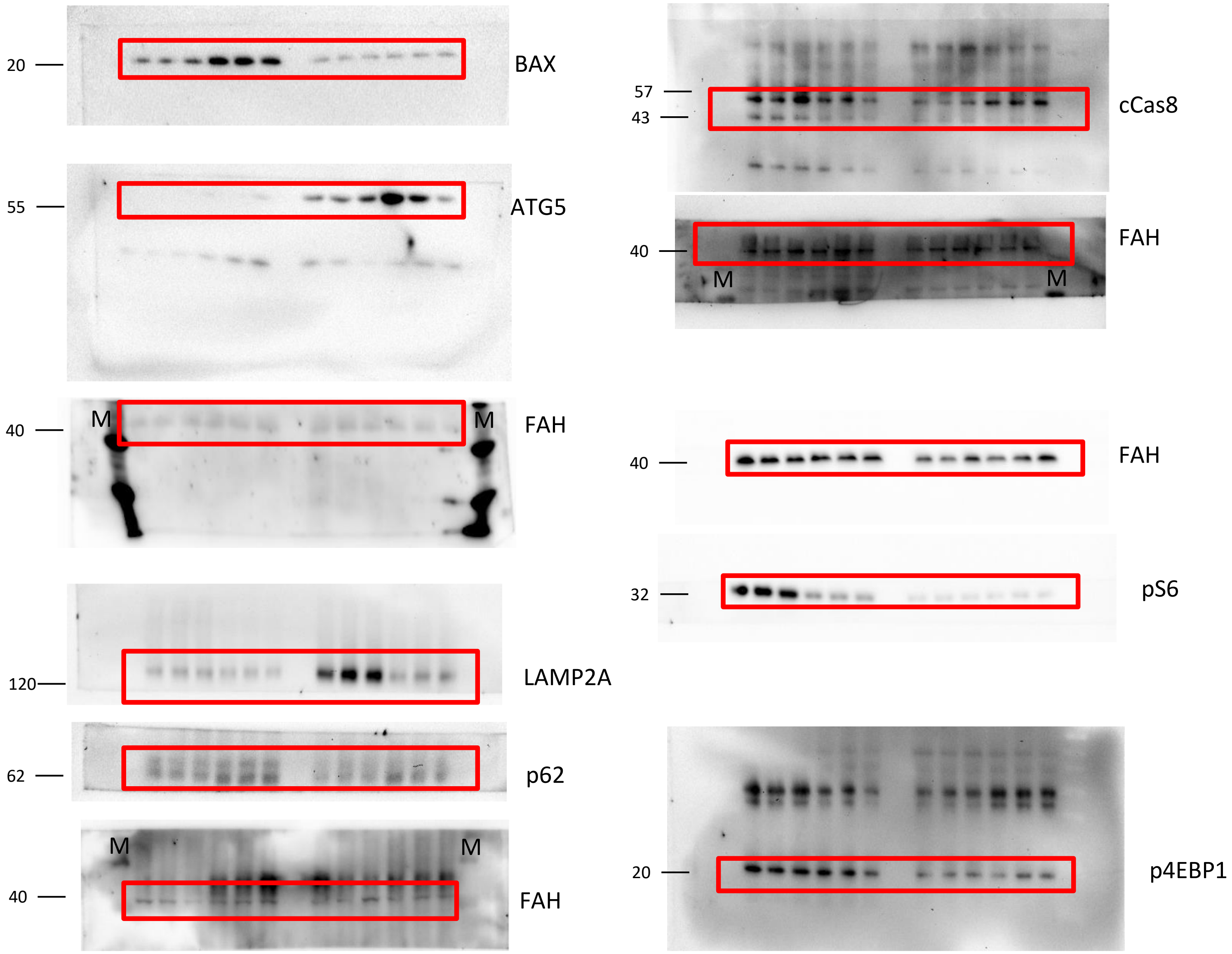

20
BAX
55
ATG5
40
M
M
FAH
120
LAMP2A
62
p62
40
M
M
FAH
57
43
cCas8
40
M
M
FAH
40
FAH
32
pS6
20
p4EBP1

Figure 5c

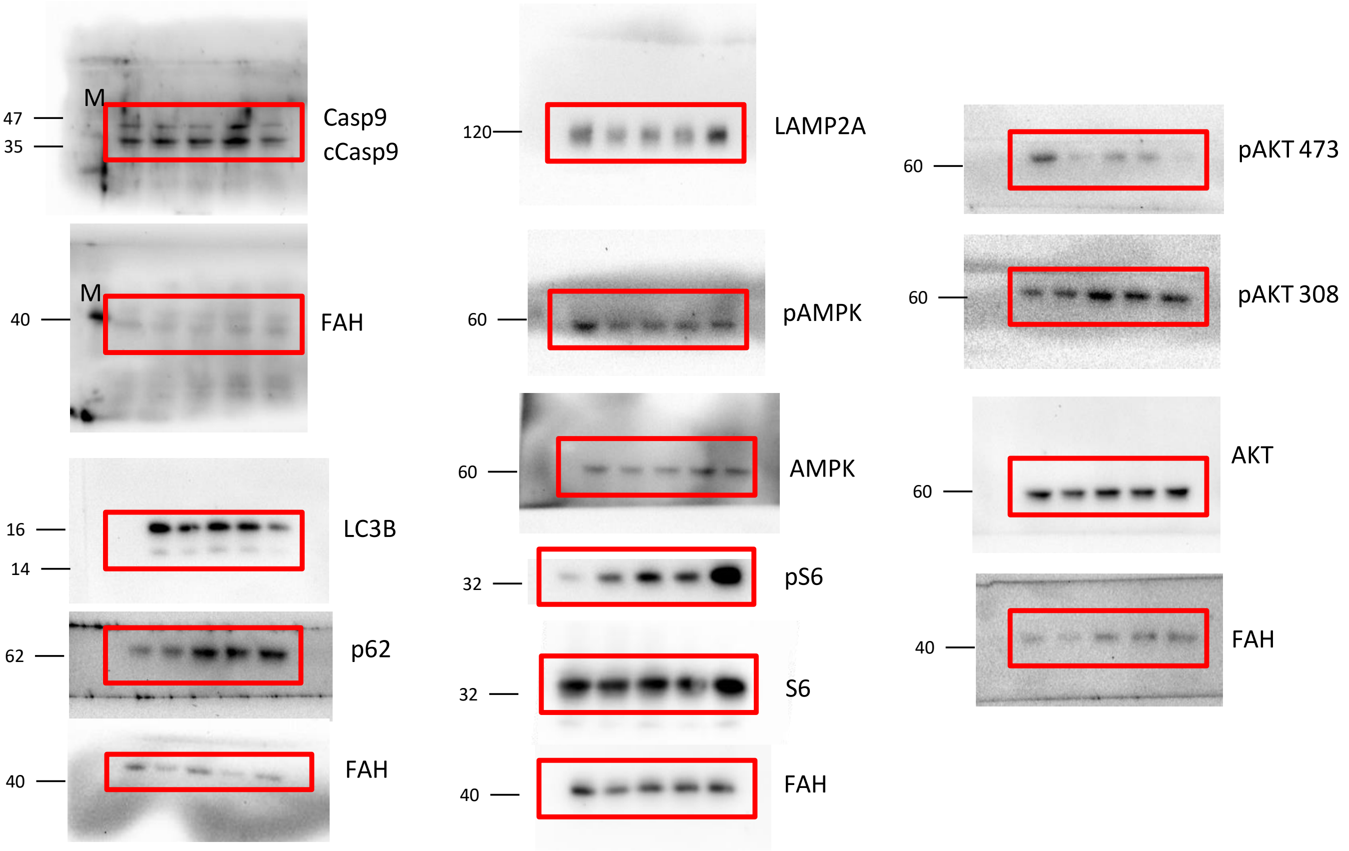

Figure 5c

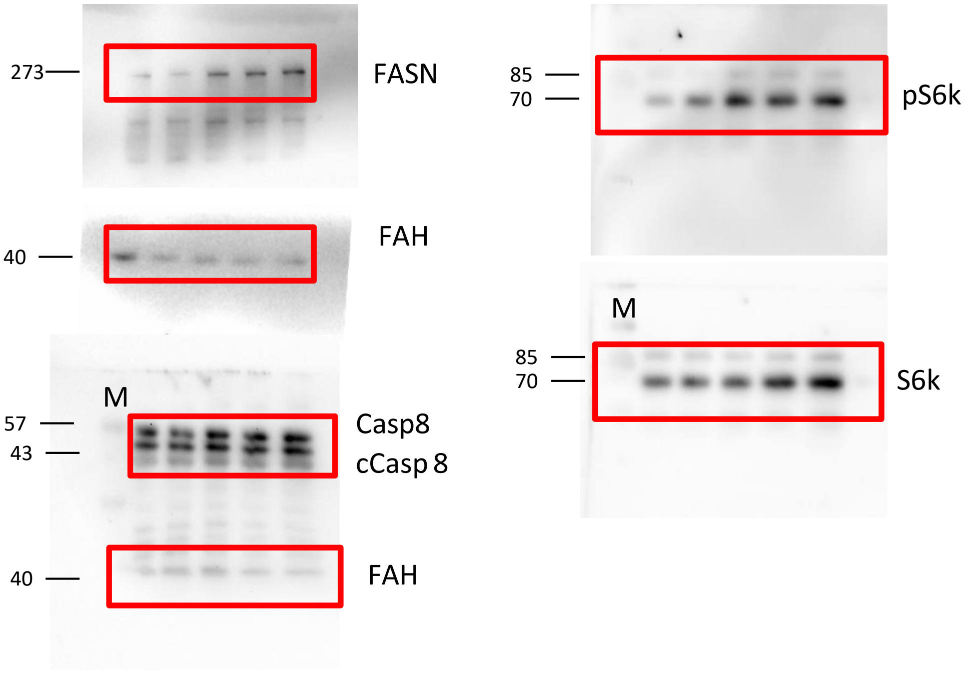

Figure 5d

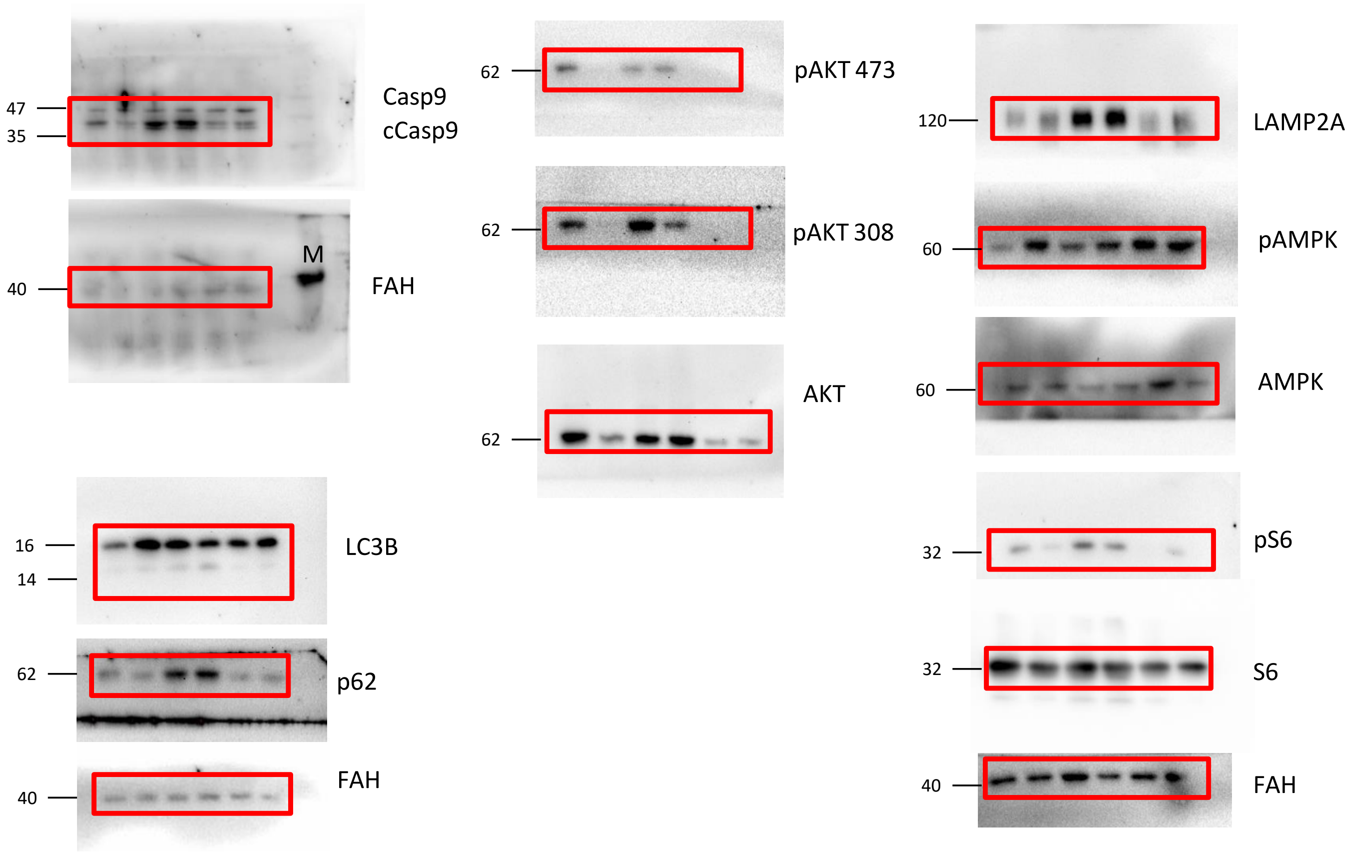

Figure 5d

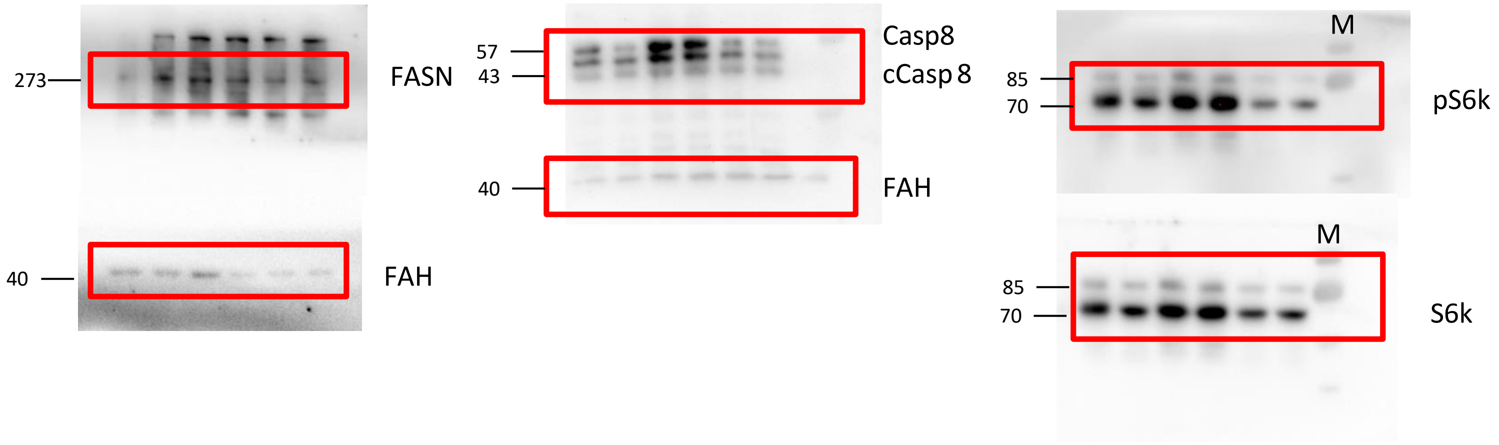

Supplementary Table S1. Clinical, laboratory assessment and liver histological features in patients with severe obesity comparing matched patients with and without NASH. NASH patients were reexamined one year after surgery

	Non-NASH (NAS ≤ 2) (n=31)	NASH (NAS ≥ 5) (n=31)	One-year after surgery (n=31)
**Clinical characteristics**			
Male, n (%)	10 (32·3)	10 (32·3)	-
Age, years	46·0 (39·0-56·0)	49·0 (44·0-56·0)	-
BMI, Kg/m^2	44·0 (41·4-46·4)	46·5 (42·6-53·6)	31·4 (28·7-33·4) [b, c]
T2DM, n (%)	11 (35·5)	17 (54·8)	4 (12·9) [b, c]
Hypertension, n (%)	17 (54·8)	21 (67·7)	10 (32·3) [b, c]
Dyslipidaemia, n (%)	9 (29·0)	12 (38·7)	2 (6·5) [b, c]
**Medication (%)**			
Metformin	4 (12·9)	12 (38·7) [a]	4 (12·9) [b, c]
Insulin	2 (6·5)	5 (16·1)	1 (3·2) [b, c]
Sulfonylureas	1 (3·2)	2 (6·5)	-
ACEIs + ARA-II	11 (35·5)	14 (45·2)	4 (12·9) [b, c]
Diuretics	4 (12·9)	5 (16·1)	-
Statins	5 (16·1)	5 (16·1)	2 (6·5) [b, c]
**Laboratory assessment**			
Hemoglobin, g/dL	13·2 (12·7-14·7)	13·4 (12·7-14·8)	13·1 (12·3-14·0)
Leukocytes, x10^9/L	7·5 (6·3-9·4)	7·6 (6·5-10·7)	6·0 (5·0-7·5) [c]
Platelets, x10^9/L	197 (187-266)	243 (181-314)	227 (209-251)
Ferritin, µg/L	44·4 (25·0-143·7)	112·4 (31·2-203·3)	32·2 (12·2-85·5) [b, c]
Total-cholesterol, mmol/L	4·3 (3·4-5·2)	4·3 (3·8-5·0)	4·8 (4·1-5·4)
HDL-cholesterol, mmol/L	1·2-0·9-1·6)	1·2 (0·9-1·4)	1·5 (1·3-1·7) [c]
LDL-cholesterol, mmol/L	2·4 (1·9-2·8)	2·6 (2·4-3·7) [a]	2·8 (2·3-3·3)
Triglycerides, mmol/L	1·5 (1·0-2·3)	1·7 (1·3-2·5) [a]	0·9 (0·6-1·4) [b, c]
Glucose, mmol/L	6·5 (6·1-8·9)	7·6 (6·2-8·7) [a]	4·5 (4·3-5·1) [b, c]
Insulin, pmol/L	90·9 (28·3-140·3)	108·0 (48·7-143·7) [a]	40·9 (20·9-58·6) [b, c]
HOMA-IR	4·1 (1·3-6·3)	6·7 (2·7-8·1) [a]	1·2 (0·6-1·9) [b, c]
Albumin, g/L	44·0 (41·0-45·0)	43·0 (41·0-46·0)	42·0 (41·0-44·0)
AST, µkat/L	0·5 (0·4-0·7)	0·7 (0·5-1·3) [a]	0·3 (0·2-0·3) [b, c]
ALT, µkat/L	0·5 (0·3-0·7)	0·7 (0·5-1·4) [a]	0·2 (0·2-0·3) [b, c]
GGT, µkat/L	0·3 (0·2-0·5)	0·5 (0·3-0·8) [a]	0·2 (0·1-0·5) [c]
CRP, mg/L	0·9 (0·5-1·5)	1·9 (0·6-1·5) [a]	0·4 (0·2-0·4) [b, c]
**Liver histologic features**			
**Steatosis**			
<5%	27 (87·1)	-	31 (100) [c]
5-33%	3 (9·7)	3 (9·7) [a]	-
34-66%	1 (3·2)	15 (48·4)	-
>66%	-	13 (41·9)	-
**Lobular inflammation**			
No foci	12 (38·7)	-	24 (77·4) [b, c]
<2 foci	16 (51·6)	5 (16·1) [a]	7 (22·6)
2-4 foci	3 (9·7)	21 (67·7)	-
>4 foci	-	5 (16·1)	-
**Hepatocellular Ballooning**			
None	26 (83·8)	2 (6·5) [a]	31 (100) [b, c]
Few cells	5 (16·1)	20 (64·5)	-
Many cells	-	9 (29·0)	-
**Fibrosis**			
None (F0)	8 (25·8)	4 (12·9)	17 (54·8) [b, c]
Perisinusoidal or periportal (F1)	18 (58·1)	8 (25·8)	13 (41·9)
Perisinusoidal and portal (F2)	5 (16·1)	15 (48·4)	1 (3·3) [b, c]
Bridging fibrosis (F3)	-	4 (12·9)	-

Values were expressed as number of cases and percentages or medians and interquartile range. ACEIs: Angiotensin-converting-enzyme inhibitor; ALT: Alanine transaminase; AST: Aspartate transaminase; ARA-II: Angiotensin II receptor antagonists; BMI: Body mass index; CRP: C-reactive

protein; HDL: High-density lipoprotein; HOMA-IR: Homeostatic model assessment of insulin resistance; HTG: Hypertriglyceridemia; LDL: Low-density lipoprotein; T2DM: Type 2 diabetes mellitus. Significant differences in comparisons are indicated by [a]non-NASH vs. NASH, [b]Non-NASH vs 12 months after surgery. [c]NASH vs one-year after surgery (at least $p<0.05$) by Wilcoxon rank-sum test.

Supplementary Table S2. Quantitative targeted metabolome in livers with, without NASH and 1 year after surgery from patients with severe obesity

	Metabolite	Non-NASH (n=31)	NASH (n=31)	One-year after surgery (n=31)
Energy Metabolism	**α-ketoglutarate**	3.5 (2.9 – 4.1)	4.4 (3.4 – 5.1) [a]	0.4 (0.3 – 0.6) [b, c]
	**β-hydroxybutyrate**	198.3 (150.5 – 247.9)	170.2 (129.7 – 208.8)	22.5 (20.2 – 27.8) [b, c]
	**Aconitate**	5.9 (4.3 – 7.5)	5.9 (4.5 – 6.5)	1.4 (1.0-2.2) [b, c]
	**Alanine**	631.7 (545.0 – 692.0)	675.5 (579.3- 727.8)	312.8 (179.8 – 553.0) [b, c]
	**Aspartate**	110.8 (73.2 – 128.2)	119.0 (84.9 – 154.7)	29.2 (23.0 – 36.4) [b, c]
	**(Iso)Citrate**	0.12 (0·07 – 0.16)	0.17 (0.11 – 0.31) [a]	0.84 (0.6 – 1.2) [b, c]
	**Fructose-1,6BP**	9.0 (7.5 – 10.2)	10.1 (8.5 – 10.8)	5.7 (3.5 – 7.5) [b, c]
	**Fructose-6P**	15.8 (13.3 – 16.6)	18.1 (15.4 – 19.7) [a]	8.9 (5.7-14.3) [b, c]
	**Fumarate**	20.8 (16.3 – 28.6)	21.0 (16.4 – 26.2)	8.4 (4.7 – 10.7) [b, c]
	**Glucose**	118.4 (75.6 – 144.8)	103.7 (81.5 – 103.7)	73.6 (37.0 – 90.9) [b, c]
	**Gluconate-6P**	9.8 (7.0 – 12.5)	7.7 (5.2 – 9.2) [a]	ND
	**Glucose-6P**	13.7 (11.4 – 15.7)	15.3 (12.9 – 16.5)	25.3 (14.8 – 30.4) [b, c]
	**Glutamate**	361.8 (242.2 – 481.8)	493.7 (351.7 – 586.7)	528.1 (359.8 – 761.7) [b]
	**Glutamine**	2212.1 (1818.1 – 2628.4)	2524.5 (1796.4 –3249.6) [a]	2110.6 (1478.9 –5212.7) [b, c]
	**Glyceraldehyde-3P**	8.3 (5.5 – 10.5)	7.2 (5.9 – 8.7)	ND
	**Glycerate-3P**	33.5 (30.2 – 47.3)	38.4 (31.6 – 54.3)	21.1 (18.5 – 21.8) [b, c]
	**Isoleucine**	209.1 (123.0 – 265.8)	208.4 (179.0 – 242.0)	12.1 (8.9 – 26.8) [b, c]
	**Lactate**	1057.0 (956.5 – 1380.7)	1085.9 (983.5 – 1212.6)	1121.2 (499.6 – 1833.9)
	**Leucine**	383.3 (245.9 – 503.2)	372.7 (301.1 – 441.6)	37.6 (30.3 – 68.7) [b, c]
	**Malate**	42.5 (35.1 – 51.3)	44.6 (34.1 – 58.5)	43.1 (27.0 – 56.0)
	**Oxolacetate**	2.3 (1.5-3.9)	2.8 (1.4-4.6)	ND
	**Phosphoenolpyruvate**	10.1 (7.5 – 15.8)	11.0 (8.7 – 13.7)	2.2 (0.6 – 2.8) [b, c]
	**Pyruvate**	7.5 (6.0 – 8.9)	10.8 (6.5 – 16.5) [a]	15.0 (6.6 – 21.0) [b, c]
	**Ribose-5P**	12.7 (9.8 – 16.3)	12.3 (11.6 – 12.4)	34.2 (23.2 – 46.4) [b, c]
	**Serine**	556.1 (338.2 – 748.9)	625.2 (538.4 – 800.2)	267.2 (155.8 – 379.0) [b, c]
	**Succinate**	25.2 (19.9 – 32.3)	25.4 (20.6 – 31.2)	62. (34.5 – 95.0) [b, c]
	**Valine**	274.3 (157.2 – 364.1)	254.1 (182.9 – 313.6)	34.0 (20.4 – 61.1) [b, c]
1-C Metabolism	**5-mTHF**	6.9 (5.3 – 12.9)	6.0 (3.1 – 9.0)	ND
	**AMP**	20.0 (9.9 – 35.1)	18.7 (10.9 – 27.5)	11.2 (4.9 – 13.7) [b, c]
	**Betaine**	26.8.8 (17.1 – 35.7)	31.1 (17.9 – 32.4)	21.3 (12.6 – 25.9) [b, c]
	**Choline**	117.5 (92.7 – 148.6)	122.7 (105.2 – 132.7)	116.3 (54.3 – 171.3)
	**Cystathionine**	0.81 (0.59 – 1.12)	0.94 (0.72 – 1.17)	0.14 (0·08 – 0.19) [b, c]
	**Cysteine**	2780.6 (1644.2 – 4776.9)	2528.9 (420.8 – 3746.7)	ND
	**Dimethylglycine**	120.8 (88.9 – 146.7)	138.7 (112.6 – 171.5)	87.2 (43.6 – 122.0) [b, c]
	**dUMP**	0.15 (0.11 – 0.21)	0.13 (0.10 – 0.18)	ND
	**Folic acid**	0·012 (0·007 - 0·017)	0·010 (0·005 - 0·014)	ND
	**Formyl-THF**	0·019 (0·012 - 0·027)	0·015 (0·011 - 0·017)	ND
	**Glycine**	481.0 (83.3 –817.4)	154.4 (79.7 –251.2) [a]	ND
	**Homocysteine**	100.7 (66.3 – 180.8)	136.6 (68.9 – 307.4)	24.2 (12.2 – 45.6) [b, c]
	**Methionine**	36.2 (24.6 – 67.1)	28.7 (19.9 – 33.3) [a]	7.23 (4.9 – 10.7) [b, c]
	**NADPH**	25.8 (17.9 – 32.8)	21.3 (14.1 – 26.3) [a]	7.2 (4.9 – 8.2) [b, c]
	**Pyridoxal-5-P**	0.21 (0.14 – 0.28)	0.25 (0.15 – 0.38)	0.18 (0.14 – 0.28)
	**Riboflavin**	0.34 (0.22 – 0.53)	0.33 (0.25 – 0.41)	0.22 (0.13 – 0.34) [b, c]
	**SAH**	12.7 (10.7 – 15.0)	9.0 (7.6 – 10.5) [a]	2.9 (1.6 – 3.7) [b, c]
	**SAM**	4.6 (2.9 – 5.5)	5.0 (3.8 – 6.9)	1.5 (0.8 – 2.7) [b, c]
	**Taurine**	9.3 (7.1 – 11.9)	12.8 (9.6 – 13.6) [a]	9.7 (5.1 – 16.3)

Data are shown as median (interquartile range) in nmol/g of tissue (dry weight). AMP, Adenosine 5′-monophosphate; dUMP, Deoxyuridine monophosphate; SAH, S-adenosylhomocysteine; SAM, S-adenosylmethionine; THF, Tetrahydrofolate· Significant differences in comparisons are indicated by [a]non-NASH vs· NASH, [b]Non-NASH vs 12 months after surgery· [c]NASH vs one-year after surgery (at least $p<0{\cdot}05$) by Wilcoxon rank-sum test.

Supplementary Table S3. The effect of supplementation with cell-permeable αKG analog (DMKG) in cultured cells

	Metabolite	Untreated (n=6)	2 Mm DMKG (n=6)	p-value
Energy Metabolism	α-ketoglutarate	0.02 (0·02 – 0·04)	0.20 (0.13 – 0.31)	**0·002**
	β-hydroxybutyrate	0.27 (0.19 – 0.36)	0.33 (0.27 – 0.52)	0·240
	Alanine	1.1 (0.89 – 1.5)	4.44 (3.36 – 7.66)	**0·002**
	Aspartate	1.78 (1.27 – 5.14)	8.6 (5.73 – 14.45)	**0·026**
	(Iso)Citrate	0.16 (0.14 – 0.23)	0.29 (0.24 – 0.44)	**0·041**
	Fructose-1,6BP	0.91 (0.67– 1.45)	1.51 (0.85 – 2.5)	0·240
	Fructose-6P	5.7 (3.58 – 7.51)	8.2 (5.0 – 11.9)	0·240
	Fumarate	0.13 (0.10 – 0.19)	0.6 (0.4 – 0.9)	**0·002**
	Glucose	10.3 (7.7 – 16.7)	8.6 (6.2 – 10.6)	0·310
	Gluconate-6P	0.23 (0.21– 0.36)	0.34 (0.24 – 0.41)	0·485
	Glucose-6P	1.7 (1.1 – 2.6)	2.6 (1.4 – 4.2)	0·240
	Glutamate	3.0 (0.8 – 6.4)	23.9 (16.9 – 38.9)	**0·002**
	Glycerate-3P	1.9 (1.1 – 3.0)	4.0 (2.2 – 6.7)	0·065
	Isoleucine	1.5 (1.3 – 2.6)	1.6 (1.6 – 1.6)	0·485
	Lactate	29.5 (24.2 – 36.2)	32.6 (24.2 – 40.9)	0·818
	Leucine	3.7 (3.2 – 4.9)	4.5 (3.3 – 5.2)	0·699
	Malate	0.28 (0.25 – 0.38)	1.15 (0.86 – 1.97)	**0·002**
	Oxaloacetate	0.8 (0.6 – 1.2)	1.7 (1.0 – 2.3)	**0·041**
	Phosphoenolpyruvate	0.19 (0·08 – 0.42)	0.36 (0.24 – 0.81)	0·240
	Pyruvate	0.14 (0.11 – 0.22)	0.28 (0.22 – 0.34)	**0·041**
	Ribose-5P	0.26 (0.14 – 0.39)	0.40 (0.3 – 0.5)	0·132
	Serine	2.0 (1.0 – 3.1)	8.1 (5.3 – 11.7)	**0·004**
	Succinate	4.6 (4.5 – 5.6)	5.4 (4.2 – 7.5)	0·485
	Valine	4.5 (3.8 – 5.3)	4.8 (3.6 – 6.0)	0·818
1-C Metabolism	AMP	0.54 (0.11 – 0.67)	0·07 (0·0 5- 0.12)	**0·004**
	Betaine*	0·008 (0·005 – 0·010)	0·003 (0·002 – 0·005)	**0·026**
	Choline-Dimethylglycine	0·05 (0·03 – 0·08)	0·02 (0·01 – 0·03)	**0·015**
	Cystathionine*	1.9 (1.8 – 3.5)	1.8 (1.5 – 1.8)	**0·015**
	Cysteine	783.3 (579.1 – 1141.8)	651.6 (635.6 – 704.3)	0·485
	Folic acid*	0.42 (0.29 – 0.54)	0.24 (0.20 – 0.40)	0·180
	Formyl-THF*	0.28 (0.24 – 0.64)	0.26 (0.11 – 0.63)	0·589
	Glycine	7.1 (5.3 – 7.5)	5.7 (4.8 – 7.9)	0·699
	Homocysteine	0·011 (0·008 – 0·014)	0·007 (0·004-0·009)	**0·041**
	Methyl-THF*	0.50 (0.30 – 0.096)	0.33 (0.22 – 0.35)	0·093
	Methylcobalamine *	0.11 (0·06 – 0.23)	0.17 (0.15 – 0.20)	0·485
	Methionine	0·036 (0·025 – 0·076)	0·038 (0·026 – 0·068)	0·485
	NADPH	0.11 (0·08 – 0.12)	0·05 (0·03 – 0·06)	**0·015**
	Pyridoxal 5-P *	3.5 (3.2 – 4.9)	2.9 (2.2 – 4.6)	0·240
	Riboflavin *	0.35 (0.34 – 0.75)	0.6 (0.4 – 0.6)	0·589
	SAH*	0·010 (0·006 – 0·023)	0·008 (0·004 – 0·009)	**0·009**
	SAM	0·071 (0·065 – 0·080)	0·058 (0·048 – 0·065)	**0·041**

Data are shown as median (interquartile range) in nmol/mg of protein except those marked with an asterisk denoting pmol/mg of protein· AMP, adenosyl monophosphate; NADPH, Nicotinamide adenine dinucleotide phosphate; SAH, S-adenosylhomocysteine; SAM, S-adenosylmethionine; THF, Tetrahydrofolate.

Supplementary Table S4. Gene expression profile in NASH livers with genes listed alphabetically according to up- and down-regulation, as compared with non- NASH livers

Upregulated

ACP5, ADCYAP1R1, AJUBA, APOL3, B3GNT5, BBC3, BHLHA15, BTG2, C12orf5, C15orf48, C2orf82, C5AR1, CAPG, CCDC109B, CCL2, CCL20, CCL3, CC[illegible]CD209, CD3G, CD52, CD83, CDCA2, CDH15, CDHR2, CFTR, CH25H, CLDN11, CLDN5, CLECL1, COL1A1, COL1A2, COL4A2-AS1, CPXM2, CPZ, CRTAM, CTSV, CXCL10, CXCL3, CXCL8, CXCL9, DOK5, DOK6, EDN2, EEF1A2, EGR2, EGR3, EZR-AS1, FABP4, FABP5, FAM151A, FAM90A7P, FAR2, [illegible]R, FFAR3, FMO1, FNDC5, FOXL2, FPR2, GATA3, GEM, GLIPR1, GPNMB, GPR182, GPR183, HIST1H1B, HIST1H3B, HLA-DQA1, HLA- DRB5, HMGCS1, HSPB8, HULC, IGSF22, IL10RB-AS1, IL1B, IL4I1, INHBE, ISM1, KCNJ3, LAMP3, LINC00884, LINC00885, LOC154872, LPL, LYPD1, MB, MB21D2, MCM2, MMP9, MNDA, NANOS3, NFKBIE, NR4A3, NTN3, OSM, PADI1, PCDH9-AS2, PEG10, PLA2G7, PLAUR, PLCXD2, PLXNC1, PODN, PRAMEF10, PROK2, PSRC1, QPCT, RASSF9, RFTN1, RGS16, RGS2, RNF186, RRAD, SEC14L3, SIX1, SLC22A13, SMIM24, SORT1, SPP1, SQLE, STMN2, TACC3, TBXAS1, THBS2, THEMIS, THY1, TIFAB, TLR9, TM4SF19, TMEM200A, TNFAIP3, TNFSF9, TREM2, TRHDE-AS1, TRIM59, TRIM63, TYMS, UGT3A2, UHRF1, UNC93A, WNT2 ,WNT5A, ZNF620, ZNF683

Downregulated

AASS, ABCA10, ACKR2, ACOT6, ADAM1A, ADCY1, ADCY10, ADHFE1, ADTRP, AFF3, AFG3L1P, AGR2, AKR1C6P, ALPK2, ANKRD23, ANO8, ARHGEF26, ARHGEF4, C1orf228, C1QTNF3, CA3, CA9, CAPN3, CATSPER3, CCDC158, CCDC162P, CCDC180, CCDC38, CCDC84, CELSR3, CENPJ, CFAP70, CHAD, CHKB, CHRD, CIART, CIT, CLASRP, CLCN2, CMYA5, COLCA2, CPT1B, CRYGS, CSPP1, CXCL2, CYP1A1, CYP3A4, CYP3A43, CYP3A5, CYP3A7, CYP4Z1, DCDC5, DCPS, DDX43, DFNB59, DGCR14, DKFZp434J0226, EFCAB1, ENO1-AS1, ERN1, FAM132A, FAM193B, FAM76B, FAM83A-AS1, FKBP5, FLJ21408, FLJ31104, FOXO1, FUT3, GADD45G, GNMT, GNRH1, GOLGA7B, GPR128, GPT2, GSTA7P, HAL, HERC2P2, HERC2P7, HERC5, HIGD1B, HORMAD2, HSD17B3, HSD3B1, ICA1, IFRD1, IGF1,IGFBP2, INS-IGF2, IRX3, ITGA10, KCNMB3, KGFLP1, KIAA0895L, KPNA7, KRT42P, KRT71, L3MBTL1, LCE2D, LGI4, LGSN, LHX4-AS1, LINC00238, LINC00659, LINC00939, LINC01125, LOC100270804, LOC100289230, LOC100505918, LOC285626, LOC644656, LOC729603, LRRC73, LYG1, MAST2, MEGF6, MREG, MT1IP, MTHFD2L, MTUS2, MYO15A, MYOM1, NBPF14, NEAT1, NEIL1, NINJ2, NNMT, NOXO1, NRBP2, OAT, P4HA1, PAPD7, PAQR6, PARP6, PATL2, PDZD3, PILRA, PILRB, POFUT2, POU6F1, PPARGC1A, PRR26, PRSS50, PSPH, PTPRH, PYROXD2, PZP, RAD51AP2, RDH12, REC8, RFPL4AL1, RHBG, RIC3, S100A1, SEC16B, SH2B1, SH2D6, SLC10A5, SLC16A1, SLC23A2, SLC25A18, SLC29A2, SLC34A1, SMIM5, SNORA33, SNORA41, SNORA6, SNORA70B, SNORA70C, SNORA70E, SN[illegible], SNORD18C, SNOR[illegible]RD98, SOCS2, S[illegible]S1R3, TAS2R19, TBC1D3B, TBC1D3C, TCAF2, TCERG1, TDRD6, TFRC, TG, TMCO6, TPTE2P5, TRPV1, TSKU, TSNAXIP1, UBE2Q2L, UCN, UGT2A1, VCPKMT, WDR60, ZDHHC11, ZNF211, ZNF266, ZNF276, ZNF507, ZNF833P

Gene ontology analysis

0

- **Cell adhesion molecules (CAMs)**
- **Chemokine signaling pathway**
- **Leukocyte transendothelial migration**
- **Lysosome**
- **Metabolism regulation**
- **Solute carrier family**
- **Small nucleolar RNA**

Supplementary Table S5. Gene expression data in NASH livers with up- and down-regulation, as compared with non-NASH patients.

Upregulated genes					
GeneName	log2FoldChange	p_value	GeneName	og2FoldChang	p_value
LINC00885	1.108266051	0.002829371	CAPG	1.3101614	0.00197551
TNFSF9	1.605596145	0.001640699	LAMP3	1.03442289	0.01288156
RNF186	1.536754651	0.047277861	FMO1	1.88537491	0.00909886
IL10RB-AS1	1.135618936	0.007232591	GEM	1.21676185	0.01258329
BHLHA15	1.119858021	0.01377422	CXCL9	1.03320944	0.00727778
ZNF683	1.161298268	0.001273694	FAR2	1.09681995	0.01731577
FPR2	1.445569009	0.008564473	MMP9	1.47015816	0.000163
HIST1H1B	1.251730534	0.040270009	PLA2G7	1.43160207	0.00188927
FOXL2	1.145449548	0.04465113	RGS16	1.45828142	0.03771505
SEC14L3	1.207605709	0.00068581	LYPD1	1.08815201	0.02034367
IGSF22	1.494253964	0.027197341	CDH15	1.39545464	0.03108971
STMN2	2.188089325	0.00933607	CCNB2	1.06475296	0.04162568
GPR183	1.271443475	0.000440736	EEF1A2	1.89120342	0.00648014
CCL2	1.634426073	0.043998403	FCAMR	1.54339159	0.0042068
RGS2	1.048396224	0.006946685	IL1B	1.94459522	0.0027467
EDN2	1.4333565	0.015961887	FAM151A	1.44265347	0.02711086
SPP1	1.214260744	0.028256747	GPNMB	1.1812359	0.02641774
NR4A3	1.375631513	0.032454529	IL4I1	1.11818242	0.00628265
FABP5	1.497196087	0.001625692	ADCYAP1R1	1.02506482	0.02922941
HIST1H3B	1.206176166	0.002622831	MB21D2	1.07487352	0.02649904
CLECL1	1.238343858	0.045466451	NTN3	1.00953129	0.01187354
COL1A2	1.140998788	0.001725005	EGR3	1.73474765	0.03692354
CPZ	1.057332488	0.037351842	UNC93A	1.09911426	0.01985908
OSM	1.57816567	0.000431245	BTG2	1.13793159	0.01114981
CCL3	1.114250666	0.024238018	CDCA2	1.00829354	0.02227709
WNT2	1.12925558	0.046624632	QPCT	1.15605831	0.00822221
GATA3	1.225255948	0.007728114	AJUBA	1.12998723	0.04753186
PADI1	3.066689154	0.002092239	EZR-AS1	1.4489299	0.0317027
CLDN5	1.021312659	0.033108993	COL1A1	1.21886276	0.00267172
SIX1	1.240592269	0.014864258	APOL3	1.0782398	0.00497664
THEMIS	1.500995323	0.000895744	PSRC1	1.06741263	0.04120535
HSPB8	1.177115944	0.018070953	RASSF9	1.08026457	0.03333617
LPL	2.481562055	0.001204697	ACP5	1.01397551	0.02173683
CXCL3	1.586512193	0.046317488	FAM90A7P	1.43185238	0.00309275
TRHDE-AS1	1.432880463	0.001455994	PODN	1.39243877	0.01026783
CCL20	1.619974764	0.033642148	TRIM59	1.0333655	0.00549998
LINC00884	1.008899566	0.030495083	CDHR2	1.25087376	0.00502943
DOK5	1.321334784	0.031174145	MB	1.84380994	0.00665582
CLDN11	1.016858101	0.011762836	SMIM24	2.30737049	0.01916011
CD3G	1.084529628	0.023614364	TLR9	1.10858318	0.02735103
MNDA	1.034074048	0.016435588	INHBE	1.13739729	0.01880832
UHRF1	1.314983104	0.002832784	COL4A2-AS	1.25129413	0.03020565
CD52	1.399552461	1.89E-05	B3GNT5	1.2871771	0.01160255
HLA-DQA1	1.746762449	0.042459273	BBC3	1.07223164	0.00516045
LOC154872	1.313600864	0.005456459	PRAMEF10	2.04642563	0.00228353
CTSV	1.16256244	0.002183877	TM4SF19	1.28956999	0.00844104
HMGCS1	1.130091553	0.001204527	CD83	1.70461926	0.0085099
NANOS3	1.252378255	0.000139541	PLXNC1	1.31395716	0.01916546
GLIPR1	1.036426354	0.010689844	FFAR3	1.5142551	0.00897662
THBS2	1.032338806	0.003884525	WNT5A	1.07413561	0.01199596
FNDC5	1.406618425	0.019345272	CCDC109B	1.13499969	0.0048924
TIFAB	1.523492563	0.008097768	PEG10	2.48542661	0.0069961
C2orf82	1.170991787	0.001451408	PLAUR	1.5661879	0.001565
SORT1	2.023260495	0.015972087	PLCXD2	1.34094042	0.02312495
UGT3A2	1.511531069	0.013210421	C5AR1	1.10317628	0.00668617
TREM2	1.797670785	0.01811032	TMEM200A	1.32201888	0.02980755
PCDH9-AS2	1.410756403	0.008774583	CH25H	2.04101754	0.03073191
ZNF620	1.165969722	0.029665648	TACC3	1.00456396	0.04173554
DOK6	1.218014335	0.010800831	CPXM2	1.35337425	0.02324825
HULC	1.79534532	0.002855902	TRIM63	1.66128199	0.01149581
C15orf48	1.132506601	0.02259033	MCM2	1.08313647	0.0026957
CRTAM	1.260530476	0.006714449	NFKBIE	1.04460348	0.01842665
TBXAS1	1.081249356	0.004787028	KCNJ3	1.05333376	0.00124271
TNFAIP3	1.021597255	0.001563517	CXCL8	2.16064077	0.01780121
CXCL10	1.58142216	0.024026638	TYMS	1.15534198	0.02740358
PROK2	1.651176691	0.008410417	THY1	1.37206997	1.32E-05
CD209	1.350897487	0.043565974	SQLE	1.6043644	0.00024769
FABP4	1.348746264	0.004523325	RRAD	1.51770217	0.02817935
HLA-DRB5	1.126678302	0.04176145	ISM1	1.47371448	0.00800775
SLC22A13	1.117547676	0.029316286	C12orf5	1.4065678	0.00551568
			CFTR	1.0345465	0.00771632
			EGR2	2.00644292	0.02349493
			RFTN1	1.18662751	0.00398026
			GPR182	1.28719289	0.00459148

Downregulated genes								
GeneName	log2FoldChange	p_value	GeneName	log2FoldChange	p_value	GeneName	log2FoldChange	p_value
WDR60	-1.126493661	0.001774784	PTPRH	-1.11299621	0.001893405	POU6F1	-1.85739276	0.000468
GADD45G	-1.947612699	0.005103815	CA9	-1.14424059	0.026604041	ARHGEF26	-1.20780671	0.009858
IGFBP2	-2.142189864	0.004480224	PRR26	-1.16557451	0.014913984	ADCY10	-2.0612402	0.005347
CRYGS	-1.368207074	0.036290713	KPNA7	-1.14681521	2.33E-05	ABCA10	-1.17408381	0.035637
PILRA	-1.323211487	0.016119236	NOXO1	-1.44398216	0.000494939	ICA1	-1.34728989	0.00753
CHAD	-1.079216635	0.008185086	EFCAB1	-1.24525592	0.003888249	CAPN3	-1.75328208	0.012313
ARHGEF4	-1.491995251	0.004623492	LOC100289230	-1.31613538	0.024469741	SYBU	-1.24599129	0.00974
DKFZp434J0226	-1.217881339	0.047275558	PAPD7	-1.13453724	0.025046828	DDX43	-1.55364884	0.00163
CYP3A5	-2.00176286	0.007499079	TCERG1	-1.05862387	0.003162446	SNORA41	-1.35308048	0.019492
SEC16B	-1.060531976	0.003476521	NNMT	-1.4945219	0.004529442	MTUS2	-1.37975481	0.035259
ACOT6	-1.054823819	0.009864916	CHRD	-1.79152394	0.027862136	ZNF276	-1.52707457	0.010258
CLCN2	-1.28755502	0.005320675	ADTRP	-1.01343989	0.00129832	PATL2	-1.0902415	0.025103
KRT71	-1.340791372	0.002811005	LGSN	-1.0940202	0.002486112	HERC2P2	-1.35455736	0.031157
FAM193B	-1.101393915	0.013508543	TCAF2	-1.18049058	0.035311117	AASS	-1.43536492	0.009894
GPT2	-1.273625123	0.00983449	FAM76B	-1.32957049	0.011563721	CA3	-1.12532466	0.006789
IGF1	-1.110791211	0.006389929	TBC1D3B	-1.7122397	0.035138425	MT1IP	-1.57448536	0.045721
CYP3A7	-1.943510904	6.42E-05	PILRB	-1.71640567	0.04625982	ANO8	-1.20068677	0.00444
CIT	-1.240458536	0.001098771	LOC100270804	-1.47292168	0.001714085	SNORA33	-1.22208689	0.017547
LYG1	-1.586894371	0.023798416	AGR2	-1.7968788	0.037994226	ZNF507	-1.43590952	0.020977
HERC2P7	-1.174357522	0.029563533	NRBP2	-1.03141893	0.006090631	SNORA70E	-1.14829444	0.041911
VCPKMT	-1.010084239	0.005192634	CCDC180	-1.61698831	0.024718146	RHBG	-1.44976652	0.014181
CATSPER3	-1.181362923	0.005549189	SLC23A2	-1.01411492	0.041434011	MEGF6	-1.01968955	0.027641
UCN	-1.042298141	0.049830757	SLC34A1	-1.34181598	0.016120516	PPARGC1A	-1.0547058	0.043285
CCDC162P	-1.361185035	0.000454615	SMIM5	-1.0234794	0.02913347	MTHFD2L	-1.35439167	0.004165
LRRC73	-1.328380079	0.009472993	TRPV1	-1.38803877	0.030937051	CENPJ	-1.02879915	0.030369
LOC100505918	-1.618054831	0.000383718	AFF3	-1.1349031	0.013133814	SNORD18C	-1.52765816	0.035507
ZDHHC11	-1.470278778	0.003716059	PAQR6	-1.23357586	0.034895557	CELSR3	-1.36441169	0.00912
KRT42P	-1.569628795	0.019052349	CSPP1	-1.03610211	0.008362508	SLC16A1	-1.06181711	0.01623
S100A1	-1.013531004	0.005872988	ZNF266	-1.02918135	0.003050812	C1orf228	-1.67870492	0.02814
RIC3	-1.39931336	0.001679276	TDRD6	-1.48727688	0.039793123	NEIL1	-1.05645288	0.015188
DFNB59	-1.609085539	0.001677429	PRSS50	-1.47990009	0.011927551	SH2D6	-1.3615019	0.018901
LINC00659	-1.194626299	0.00017105	CXCL2	-1.39777064	0.033398815	TSKU	-1.05789448	0.000901
UBE2Q2L	-1.084857159	0.032211555	SH2B1	-1.00707108	0.010187832	CYP4Z1	-1.6292643	0.005059
ADCY1	-1.03533066	0.009044822	CMYA5	-1.88961881	0.004205976	ITGA10	-1.85265283	0.007251
TSNAXIP1	-1.244770891	0.007667743	P4HA1	-1.72685563	0.000608092	C1QTNF3	-1.1330806	0.020593
NEAT1	-2.100398711	0.02309016	HSD17B3	-1.26045501	0.049483051	OAT	-1.30476489	0.015518
SNORA72	-1.274518492	0.022261622	FLJ21408	-1.70077327	0.025790691	KIAA0895L	-1.65475067	0.047397
FKBP5	-1.425452795	0.047662255	RFPL4AL1	-1.18552897	0.046163437	AFG3L1P	-1.33548026	0.045384
ZNF211	-1.251199378	0.016830068	CIART	-1.15097359	0.047524274	CYP1A1	-1.75334242	0.011345
GOLGA7B	-1.17791694	0.028403328	LINC01125	-1.44058468	0.034571851	HAL	-1.57754608	0.02019
CCDC84	-1.15751599	0.018016694	SOCS2	-1.71615046	0.000992992	GNMT	-1.16873572	0.015956
GNRH1	-1.007087074	0.021117891	FAM132A	-1.16590025	0.029318286	SNORA70C	-1.49826649	0.029462
ADHFE1	-1.129910177	8.13E-05	KCNMB3	-1.33571172	0.032448127	SLC10A5	-1.29351273	0.002953
TAS1R3	-1.204752139	0.026546907	LOC644656	-1.25457553	0.036935149	KGFLP1	-1.11942506	0.027451
LINC00238	-1.352417355	0.000206214	PARP6	-1.00612529	0.038261869	SLC25A18	-1.67830532	0.018477
TG	-1.362292804	0.001616555	ERN1	-1.15768826	0.025821033	ALPK2	-1.03852867	0.04912
UGT2A1	-1.655474606	0.047696138	TAS2R19	-1.51169392	0.040660052	LOC729603	-1.23185661	0.011661
SLC29A2	-1.006325302	0.003901491	HERC5	-1.00498747	0.000740968	REC8	-1.11254282	0.030743
CCDC158	-1.456799089	0.020690154	FLJ31104	-1.17486704	0.007729886	CYP3A43	-2.31859675	0.002867
CPT1B	-1.670810078	0.0436173	DCPS	-1.26871742	0.019435557	PSPH	-1.87290395	0.012305
AKR1C6P	-1.280129373	0.014501026	LINC00939	-1.55396493	0.041503613	ENO1-AS1	-1.45557871	0.005486
MAST2	-1.125526239	0.016840142	DCDC5	-1.77184007	0.004577131	ACKR2	-1.09748842	0.017915
GSTA7P	-1.714817218	2.46E-05	HSD3B1	-1.14440667	0.014152964	RDH12	-2.07750891	0.00122
LHX4-AS1	-1.371608943	0.032521631	PYROXD2	-1.0742378	0.015932891	PDZD3	-1.08890161	0.026144
FUT3	-1.86470039	0.013011228	SNORA6	-1.61078428	0.046323089	ZNF833P	-1.13906821	0.03171
LOC285626	-1.486953604	0.002049649	HORMAD2	-1.43334661	0.001193126	TPTE2P5	-1.13779005	0.002817
GPR128	-1.354022024	0.007686289	DGCR14	-1.03707075	0.025475074	LCE2D	-1.0390637	0.022922
CYP3A4	-1.242529448	0.005431129	FOXO1	-1.02089662	0.002522249	ADAM1A	-1.01050249	0.039817
L3MBTL1	-1.004317846	0.047856923	TBC1D3C	-2.03687695	0.015344298	INS-IGF2	-1.32319727	0.02428
CCDC38	-1.51983207	0.027374977	RAD51AP2	-1.00284032	0.043864866	IRX3	-1.56592518	0.000566
POFUT2	-1.073197128	0.018255897	TMCO6	-1.26949896	0.00020521	SNORD85	-1.08310005	0.013902
SNORA70B	-1.078813343	0.04286567	COLCA2	-1.05685019	0.020575812			
CFAP70	-1.556497279	0.007343097	IFRD1	-1.06333905	0.019435381			
PZP	-1.537947401	0.021468382	SNORD98	-1.08777867	0.027374871			
MREG	-1.199152475	0.01863969	HIGD1B	-1.00103968	0.025156285			
NBPF14	-1.335820315	0.022385849	CHKB	-1.04593312	0.037667327			
ANKRD23	-1.040173325	0.00755223	MYO15A	-1.82349261	0.00942321			
TFRC	-1.244119166	0.022097264	NINJ2	-1.03573936	0.002018139			
CLASRP	-1.127514006	0.030205002	LGI4	-1.80790558	0.021603778			
FAM83A-AS1	-2.128722729	0.006993813	MYOM1	-1.39427805	0.014236142			

Supplementary Table S6. List of altered CpG sites in NASH livers, as compared with non- NASH livers

Gene Name	CpG	Gene Name	CpG	Gene Name	CpG	Gene Name	CpG	Gene Name	CpG
A2ML1	cg12168926	C1orf54	cg06334965	CYP2C19	cg24857560	FGD4	cg11809958	HMOX2	cg02873098
ABCC3	cg18891141	C20orf78	cg19205181	CYP7A1	cg15805923	FGF22	cg23518439	HNF4A	cg23834593
ACP5	cg01524690	C2orf54	cg20140201	DCC	cg02835371	FGFR1	cg15791248	HNRNPKP3	cg01405920
ACTN1	cg04425263	C5orf56	cg03168315	DEFB119	cg13817555	FKBP5	cg21517946	IDH3B	cg27161589
ADAMDEC1	cg18212777	C5orf66	cg25768573	DEFB121	cg13795252	FKBP5	cg21517946	IGF2	cg08686462
ADAMTS14	cg26971530	CAD	cg25258016	DEFB126	cg01745599	FGF22	cg23518439	IGSF8	cg12390003
ADAMTS16	cg07834743	CARD10	cg16026346	DEFB132	cg08841021	FGFR1	cg15791248	IKZF1	cg01139861
ADIG	cg13816419	CCDC122	cg13487505	DHRS7B	cg19360316	FKBP5	cg21517946	IL32	cg20877076
AKR1C4	cg06573570	CCDC129	cg23825123	DHRS7B	cg05694098	FLG	cg19855573	IPCEF1	cg19690214
AMPH	cg14920797	CCDC144NL	cg22570042	DISP2	cg17063840	FOXI1	cg07849278	ISM1	cg15577927
ANKRD34C-AS1	cg15981071	CCDC15	cg20250779	DLGAP1	cg25358264	FOXO1	cg02202169	ITGA4	cg25515269
ANKS1B	cg18299299	CCDC166	cg08435853	DNAAF3	cg09204255	FYN	cg08061598	IYD	cg08400967
APH1B	cg09264088	CCDC166	cg14540594	DOC2A	cg19699287	GABARAP	cg16879549	KCNA3	cg00995520
APOBEC2	cg09841052	CCDC59	cg03652173	DOCK10	cg19407684	GALNT1	cg16741573	KCNK10	cg01523348
ARHGEF4	cg09015973	CCT8L2	cg02601618	DPYSL3	cg13669166	GAPDHS	cg15729137	KCNQ1	cg23623667
ARL4A	cg07227033	CDC23	cg24961402	DUSP1	cg06819445	GATM	cg11032707	KDM5B	cg08718872
ARL8A	cg08649954	CEACAM21	cg04946843	ECHDC2	cg06600287	GBA3	cg17751101	KIAA0319	cg18433519
ARPP21	cg10504927	CHI3L1	cg14085262	EEF1D	cg14824382	GGA2	cg05579124	KIAA0319	cg12349317
ARSK	cg11569235	CHMP6	cg00774579	EFHD1	cg17021917	GGPS1	cg10107779	KIAA0319	cg00944873
ATCAY	cg08000406	CHST8	cg24221853	EIF2AK2	cg23820429	GPLD1	cg02346062	KIAA0319	cg16906346
ATG101	cg18315870	CHST9	cg01960885	EIF4ENIF1	cg01782953	GPR160	cg14168775	KIAA1191	cg26620356
ATG101	cg05628902	CLDN18	cg20366986	ELAC1	cg20726616	GPR75	cg20081082	KIF14	cg22888029
ATP5A1	cg26626598	CLEC18B	cg27583026	ELAVL4	cg25217583	GPS2	cg12391328	KLB	cg06321636
ATP6V0E2	cg18934443	CLEC3B	cg22505962	ELAVL4	cg09784523	GRAMD3	cg06609646	KLF5	cg20314763
ATP6V1G1	cg14391907	CLIC3	cg23224755	ELF1	cg15040708	GSDMB	cg10057218	KLK8	cg13388253
BATF	cg21158815	CLVS2	cg20134787	ELOVL3	cg24362923	GSX1	cg10071824	LDHAL6A	cg06888460
BCAT1	cg20783223	CMTM3	cg12370353	ENPP3	cg12121052	GTF2A2	cg00368989	LGALS8	cg04027302
BCL7A	cg10552903	CNBP	cg02658964	ESR1	cg20583095	GTF2IRD1	cg07645228	LILRA2	cg23089880
BCL7C	cg13914857	CNGA3	cg00209520	ETNK1	cg21477262	GULP1	cg08913189	LIMK2	cg01606027
BCL9L	cg20029201	CNNM1	cg06312469	EXPH5	cg14140118	GZMA	cg13566919	LINC01146	cg24014326
BDNF	cg06260077	CNTN1	cg24360230	FABP12	cg18190353	H19	cg06658831	LINC01168	cg15681295
BIN1	cg02161262	COLEC12	cg21067023	FAIM3	cg17460386	HAO1	cg01478327	LOC441666	cg24527560
BOLL	cg10547527	COX7A2	cg25716013	FAM118A	cg00733150	HARS2	cg26273129	LPPR1	cg04778212
BOLL	cg06077337	CR1	cg25029035	FAM193B	cg18478850	HAS3	cg09944073	LRRC10B	cg06964190
BRCA1	cg14947218	CRYBG3	cg22429199	FAM222A	cg14544673	HCST	cg16001418	LRRC55	cg03717588
BRCA1	cg02286533	CRYZL1	cg16762072	FAM60A	cg01611280	HDAC9	cg19585556	LRRTM1	cg26157270
C17orf98	cg18343108	CTSV	cg24546976	FBXO34	cg18427465	HIBCH	cg06484496	LRRTM3	cg09455274
C17orf98	cg12552293	CYB5R3	cg18535283	FCRLA	cg05033369	HIST1H2BE	cg22942804	LSP1	cg15348841
C19orf84	cg11030887	CYP2C19	cg27505447	FERMT1	cg25325723	HLF	cg25534244	LTBR	cg10452531
**Gene Name**	**CpG**	**Gene Name**	**CpG**	**Gene Name**	**CpG**	**Gene Name**	**CpG**	**Gene Name**	**CpG**
MAD2L1	cg14360823	OR7D4	cg04487907	RGS14	cg15591490	SPSB2	cg09080114	VMO1	cg24874612
MAGOHB	cg03208983	OSMR	cg19609242	RGS14	cg11370586	ST3GAL1	cg02637438	VPS39	cg14774557
MAL2	cg02085119	P2RX6	cg09854409	RGS4	cg20056654	ST6GALNAC2	cg21914725	WBP2NL	cg08431931
MANSC4	cg13312247	P2RX7	cg08688169	RGS5	cg17683793	ST6GALNAC4	cg13886636	WBP2NL	cg04717802
MARK3	cg26623547	PAK1IP1	cg12409563	RIBC2	cg18180155	STMN2	cg13068285	WBP2NL	cg25099233
MARS	cg06714758	PCDHA2	cg27546766	RIBC2	cg05742863	STT3A	cg19571034	WISP1	cg13358979
MBIP	cg12645564	PCDHA8	cg02326566	RIBC2	cg00610021	SUSD5	cg03497399	WNT3	cg11142826
MED9	cg00723891	PCDHB12	cg17101542	RNF14	cg21256257	SYNJ1	cg18548246	WNT9A	cg05183226
MFAP1	cg12801082	PCDHB12	cg15548198	RNF214	cg04174309	TAB2	cg06416086	YAP1	cg12697442
MGAT4A	cg14223723	PCDHB2	cg23969338	RNF25	cg26548497	TAGLN	cg11861562	ZBTB48	cg19508900
MGC27382	cg13296396	PCDHGB4	cg22648135	RNPS1	cg08301299	TARSL2	cg10526733	ZCCHC4	cg13975540
MICAL3	cg06200397	PCP2	cg03489492	RORB	cg14503744	TBC1D1	cg06331663	ZFR2	cg08098382
MOBP	cg27272723	PDE4B	cg10277218	RPA3	cg26322315	TDRD6	cg08426066	ZNF197	cg11557071
MORF4L1	cg20596706	PDE4D	cg04545873	RPS6KL1	cg12643970	TDRD6	cg11931223	ZNF260	cg24909706
MRPL34	cg01445659	PEX2	cg22944540	RSPH6A	cg02505812	THAP1	cg20173334	ZNF287	cg12597066
MRPS10	cg24613956	PIAS3	cg15978276	RSPH6A	cg07377422	TMCO1	cg10234330	ZNF474	cg18554917
MTAP	cg23850567	PKIA	cg04689061	SAMD3	cg09345868	TMEM105	cg21591452	ZNF527	cg19000959
MVB12A	cg02608002	PLCD4	cg18771537	SCARNA6	cg24516399	TMEM105	cg06727703	ZNF883	cg02199333
MYO1C	cg12741184	PLIN1	cg08749443	SCML4	cg08792995	TMEM132E	cg21745612	ZP2	cg18249108
MYOCD	cg18181954	POTEA	cg01192262	SEC11C	cg20600053	TMEM194A	cg01217666		
NCCRP1	cg18627816	PPBP	cg20543211	SENP1	cg12381317	TMEM200B	cg15307678		
NDUFAF3	cg07109801	PPM1A	cg14785542	SETD9	cg06961054	TMEM200B	cg22788029		
NDUFB10	cg07623113	PSG6	cg20120646	SETD9	cg06795995	TMEM217	cg18131329		
NKX6-2	cg17996619	PTCHD3	cg09544380	SHD	cg24830664	TMEM217	cg00480799		
NLRP6	cg09205751	PTH2	cg22250242	SHPK	cg21817720	TNFAIP8L2	cg22392857		
NOSIP	cg06468619	PTPN7	cg01520554	SIRT6	cg05075545	TRAPPC1	cg10518440		
NRON	cg09518226	PWWP2A	cg20433356	SLC10A5	cg20769419	TRIM7	cg16027343		
NUDT21	cg14837652	PYCARD	cg09115984	SLC15A5	cg26345444	TRIM8	cg00793774		
OLFM3	cg16510022	RAB1B	cg25618573	SLC29A2	cg07183539	TRIP10	cg18732869		
OLFML2A	cg25612362	RAB1B	cg04592747	SLC6A4	cg06841846	TRIT1	cg04921771		
OR14I1	cg25289056	RAB1B	cg15201417	SLC7A8	cg21380590	TRPC6	cg07898145		
OR1A2	cg03271520	RAB31	cg04995377	SMG6	cg27337217	TSACC	cg19759212		
OR2L8	cg16077391	RAB37	cg08080145	SNCB	cg25444839	TWIST1	cg14391419		
OR2L8	cg18857759	RALGAPB	cg15971382	SND1	cg04345581	TXNL1	cg00152799		
OR4D1	cg12463722	RANBP3	cg08371934	SND1	cg11539674	TYW3	cg12981534		
OR4D1	cg20221991	RAPGEF1	cg15815515	SNORA71C	cg11363483	UCHL3	cg07830652		
OR4D2	cg24269480	RASSF5	cg16122716	SNX13	cg06049192	UGT3A2	cg10402936		
OR51B2	cg12036731	RBKS	cg16651262	SPDYC	cg03196265	UTS2	cg15727920		
OR51Q1	cg05975928	RET	cg01289552	SPRR2C	cg07804289	VIL1	cg18970338		

Supplementary Table S7. List of antibodies and dilutions used in immunoblot analyses

Antigen	Primary Antibody	Dilution	Secondary Antibody	Dilution
**4-HNE**	4-HNE antibody, #393206 (Millipore)	1:1000	Goat α-rabbit HRP, P0448 (Dako Agilent, Santa Clara CA)	1:5000
**Actin**	Pan-Actin antibody, #4968 (Cell signalling, Danvers, MA)	1:10000	Goat α-mouse, HRP, 1D3 (Dako Agilent, Santa Clara CA)	1:5000
**AMPK-pT172**	pAMPK Antibody, #2531 (Cell signalling, Danvers, MA)	1:1000	Goat α-rabbit HRP, P0448 (Dako Agilent Santa Clara CA)	1:5000
**AMPK**	AMPK Antibody #2532S (Cell signalling, Danvers, MA)	1:1000	Goat α-rabbit HRP, P0448 (Dako Agilent, Santa Clara CA)	1:5000
**AKT-pT308**	p-Akt Antibody, #4056 (Cell signalling, Danvers, MA)	1:1000	Goat α-rabbit HRP, P0448 (Dako Agilent, Santa Clara CA)	1:5000
**AKT-pT473**	p-Akt Antibody, #4060 (Cell signalling, Danvers, MA)	1:1000	Goat α-rabbit HRP, P0448 (Dako Agilent, Santa Clara CA)	1:5000
**AKT**	AKT Antibody, #4685 (Cell signalling, Danvers, MA)	1:1000	Goat α-rabbit HRP, P0448 (Dako Agilent, Santa Clara CA)	1:5000
**ATG5**	ATG7 Antibody, #12994 (Cell signalling, Danvers, MA)	1:1000	Goat α-rabbit HRP, P0448 (Dako Agilent, Santa Clara CA)	1:5000
**mTOR-pS2448**	p-mTOR Antibody, #2971 (Cell signalling, Danvers, MA)	1:1000	Goat α-rabbit HRP, P0448 (Dako Agilent, Santa Clara CA)	1:2000
**mTOR**	mTOR Antibody, #2972 (Cell signalling, Danvers, MA)	1:200	Goat α-rabbit HRP, P0448 (Dako Agilent, Santa Clara CA)	1:2000
**S6-pS235/236**	p-S6 Antibody, #4856 (Cell signalling, Danvers, MA)	1:1000	Goat α-rabbit HRP, P0448 (Dako Agilent, Santa Clara CA)	1:5000
**S6**	S6 Antibody, #2217 (Cell signalling, Danvers, MA)	1:1000	Goat α-rabbit HRP, P0448 (Dako Agilent, Santa Clara CA)	1:5000
**S6K-pS235/236**	p-S6K Antibody, #9205 (Cell signalling, Danvers, MA)	1:1000	Goat α-rabbit HRP, P0448 (Dako Agilent, Santa Clara CA)	1:5000
**S6K**	S6K Antibody, #2708 (Cell signalling, Danvers, MA)	1:1000	Goat α-rabbit HRP, P0448 (Dako Agilent, Santa Clara CA)	1:5000
**BAX**	BAX Antibody, #5023 (Cell signalling, Danvers, MA)	1:1000	Goat α-rabbit HRP, P0448 (Dako Agilent, Santa Clara CA)	1:5000
**4EBP1-pT37/46**	p-4E-BP1 Antibody, #2855 (Cell signalling, Danvers, MA)	1:1000	Goat α-rabbit HRP, P0448 (Dako Agilent, Santa Clara CA)	1:5000
**p62/SQSTM1**	SQSTM1 / p62 Antibody, #5114 (Cell signalling, Danvers, MA)	1:1000	Goat α-rabbit HRP, P0448 (Dako Agilent, Santa Clara CA)	1:5000
**Caspase 3**	Cleaved Caspase-3 Antibody, #9664 (Cell signalling, Danvers, MA)	1:1000	Goat α-rabbit HRP, P0448 (Dako Agilent, Santa Clara CA)	1:5000
**Caspase 8**	Caspase-8 Antibody, #9746 (Cell signalling, Danvers, MA)	1:1000	Goat α-rabbit HRP, P0448 (Dako Agilent, Santa Clara CA)	1:5000
**Caspase 9**	Caspase-9 Antibody, C7729 (Sigma, Saint Louis, MO)	1:1000	Goat α-rabbit HRP, P0448 (Dako Agilent, Santa Clara CA)	1:5000
SAPK/Jnk-p	SAPK/JNK-p Antibody, #9251 (Cell signalling, Danvers, MA)	1:1000	Goat α-rabbit HRP, P0448 (Dako Agilent, Santa Clara CA)	1:5000
MAK (Erk1/2) p-P44/42	p-P44/42 ERK1/2 Antibody, #9101 (Cell signalling, Danvers, MA)	1:1000	Goat α-rabbit HRP, P0448 (Dako Agilent, Santa Clara CA)	1:5000
MAPK (Erk1/2) P44/42	P44/42 ERK1/2 Antibody, #9102 (Cell signalling, Danvers, MA)	1:1000	Goat α-rabbit HRP, P0448 (Dako Agilent, Santa Clara CA)	1:5000
**IL10**	IL10 antibody, ab34843 (Abcam, Cambridge, UK)	1:1000	Goat α-rabbit HRP, P0448 (Dako Agilent, Santa Clara CA)	1:5000
**MFN2**	MFN2 Antibody, ab127773 (Abcam)	1:1000	Goat α-rabbit HRP, P0448 (Dako Agilent, Santa Clara CA)	1:5000

NFKB	NFKB p65 Antibody, #8242 (Cell signalling, Danvers, MA)	1:1000	Goat α-rabbit HRP, P0448 (Dako Agilent, Santa Clara CA)	1:5000
**OXPHOS**	OXPHOS Antibody, ab110411 (Abcam)	1:250	Goat α-mouse, HRP, 1D3 (Dako Agilent, Santa Clara CA)	1:5000
p38	p-p38 MAPK Antibody, #9211 (Cell signalling, Danvers, MA)	1:1000	Goat α-rabbit HRP, P0448 (Dako Agilent, Santa Clara CA)	1:5000
**STAT3-pT705**	p-STAT3 Antibody, #9145 (Cell signalling, Danvers, MA)	1:1000	Goat α-rabbit HRP, P0448 (Dako Agilent, Santa Clara CA)	1:5000
**STAT3**	STAT3 Antibody, #9139 (Cell signalling, Danvers, MA)	1:1000	Goat α-mouse HRP, P0447 (Dako, Agilent, Santa Clara CA, USA)	1:5000
**LC3B**	LC3B Antibody, #2775S (Cell signalling, Danvers, MA)	1:1000	Goat α-rabbit HRP, P0448 (Dako Agilent, Santa Clara CA)	1:5000
**LAMP2A**	LAMP2A Antibody, ab125068 (Abcam)	1:1000	Goat α-rabbit HRP, P0448 (Dako Agilent, Santa Clara CA)	1:5000
**FASN**	FASN Antibody, #3180 (Cell signalling, Danvers, MA)	1:1000	Goat α-rabbit HRP, P0448 (Dako Agilent, Santa Clara CA)	1:5000
**TOM20**	Tom20 Antibody, #42406 (Cell signalling, Danvers, MA)	1:1000	Goat α-rabbit HRP, P0448 (Dako Agilent, Santa Clara CA)	1:5000
**MFN2**	MFN2 Antibody, ab127773 (Abcam)	1:1000	Goat α-rabbit HRP, P0448 (Dako Agilent, Santa Clara CA)	1:5000
**FAH**	FAH Antibody, #ABN526 (Millipore, Massachusetts, MA)	1:1000	Goat α-rabbit HRP, P0448 (Dako Agilent, Santa Clara CA)	1:5000
**GADPH**	GADPH Antibody, #2118 (Cell signalling, Danvers, MA)	1:8000	Goat α-rabbit HRP, P0448 (Dako Agilent, Santa Clara CA)	1:5000

Supplementary Table S8. List of reagents and procedures provided via Taqman® gene expression assays used in quantitative RT-PCR. The expressions of housekeeping gene 18S (Hs03928985_g1) were used for calculations

Gene expression assay	Gene name
**αKGDH** Hs01081865_m1	Alpha-Ketoglutarate Dehydrogenase
**ACACA** Hs01046047_m1	Acetyl-CoA Carboxylase Alpha
**ACLY** Hs00153764_m1	ATP citrate lyase
**IDH1** Hs00271858_m1	Isocitrate Dehydrogenase 1
**IDH2** Hs00953881_m1	Isocitrate Dehydrogenase 2
**IDH3A** Hs00194253_m1	Isocitrate Dehydrogenase 3A
**GSL1** Hs00248163_m1	Glutaminase
**GLUD1** Hs03989560_s1	Glutamate Dehydrogenase 1
**PC** Hs00559398_m1	Pyruvate Carboxylase
**SDHB** Hs01042482_m1	Succinate Dehydrogenase B
**ACP5** Hs00356261_m1	Acid Phosphatase 5, Tartrate Resistant
**ARL8A** Hs00373395_m1	ADP Ribosylation Factor Like GTPase 8A
**C1orf54** Hs04398113_m1	Chromosome 1 Open Reading Frame 54
**DISP2** Hs00394338_m1	Dispatched RND Transporter Family Member 2
**HDAC9** Hs00206843_m1	Histone Deacetylase 9
**MARK3** Hs01058270_m1	Microtubule Affinity Regulating Kinase 3
**RAB31** Hs00199313_m1	RAB31, Member RAS Oncogene Family
**TDRD6** Hs01597145_m1	Tudor Domain Containing 6
**TRIP10** Hs01012747_m1	Thyroid Hormone Receptor Interactor 10
**UGT3A2** Hs04177793_m1	UDP Glycosyltransferase Family 3 Member A2
**ZNF197** Hs01560359_m1	Zinc Finger Protein 197

Supplementary methods

Extended Study design and participants

Patients are included consecutively after previous failure in management after dietary treatments, changes in lifestyle factors and psychological assessment. We matched as closely as possible patients with and without NASH, and NASH patients agreed to have a second liver biopsy at 1 year after successful surgery. Before surgery, the drug schedule was revised and some were interrupted, especially metformin, to avoid described side effects. Some drugs were again prescribed after surgery according to the clinical follow-up. Some variables depicted in Table S8 indicated that weight loss and improvement of glucose and lipid metabolism were fully achieved during the first year. When necessary, healthy non-obese controls were used as described. [1] Clinical indication for laparoscopic sleeve gastrectomy (LSG) was according to guidelines currently used in pre-operative evaluation. We excluded patients with current, or past, history of daily alcohol abuse (≥ 30 g for men and ≥ 20 g for women), long-term consumption of hepatotoxic drugs, and liver disease of infectious origin. LSG was performed under general anaesthesia with the patient in the Lloyd-Davies position. A five-port technique was used in all patients. The greater gastric curvature was dissected, separated from the gastroepiploic arcade of the greater omentum, and continued to the His angle. The gastric transection was performed under the guidance of a 38-Fr Faucher bougie. Three cm was the distance from the pylorus to the first section point. The suture line was reinforced using polycarbonate derivatives of polyglycolic acid to avoid haemorrhages and leaks. A methylene blue leak was always performed before closing abdominal wall. [2,3]

Transmission electron microscopy

Small pieces of the liver were first fixed in a 2% glutaraldehyde solution (pH 7·4), washed with 0·1 M cacodylate and then fixed with osmium tetroxide. Thick sections stained with 1% toluidine blue were used to identify the area of interest, and ultra thin sections (70 nm) were used for examination after staining with uranyl acetate and lead citrate.

Immunoblotting analysis and quantification

The uncropped blots corresponding to those presented in the Figures of the main article are shown in Figure S13. Homogenates for western blot analyses were obtained from either the liver samples or cell cultures. The proteins were resolved in 8% or 14% polyacrylamide gels by SDS-PAGE and transferred to polyvinylidene difluoride or nitrocellulose membranes (Thermo Fisher, Barcelona, Spain). The sources and dilutions of the primary and secondary antibodies may be found in Table S7. The immunoreactive bands were visualised using the SuperSignal West Femto chemiluminescent substrate (Pierce, Rockford, IL, USA), and the analysis was performed with a ChemiDoc system (Bio-Rad Laboratories, Madrid, Spain). Fumarylacetoacetate hydrolase (FAH) is used as a loading control. The bands were analysed and quantified using Image Lab 2.0 software (Bio-Rad Laboratories). All WB quantifications were normalized with the housekeeping FAH or by the ratio of total protein vs. phosphorylated protein. Phosphorylated proteins (pAKT, pAMPK, pMTOR, pS6k, and pS6) were developed and then stripped to determine total proteins. To remove primary and secondary antibodies we used 62.5 mM Tris-HCl, pH 6.7 buffer with 100 mM b-mercaptoethanol, and 2% SDS. We incubated 15 minutes at 50°C and then we washed the membranes with TBST 5 minutes 10 times before blocking.

Immunocytochemistry

After the deparaffinisation and rehydration of the liver sections, the antigens were retrieved in 0·15 M sodium citrate buffer at pH 6 in a microwave oven up to 90°C. Immunocytochemistry was performed using antibodies against 4-hydroxy-2-nonenal and paraoxonase-1. The cells stained for specific markers were quantified using ImageJ software (National Institutes of Health, Bethesda, MD, USA). The percentage of apoptotic nuclei in liver biopsies was determined by a colorimetric terminal deoxynucleotidyl transferase-mediated dUTP nick end labelling (TUNEL) assay (Click-iT™ TUNEL, Invitrogen, Thermo Fisher, Waltham, MA, USA) according to the manufacturer's instructions.

Quantitative targeted metabolomics platforms

To measure the metabolites from energy metabolism, we used a 7890A gas chromatograph coupled with an electron impact source to a 7200-quadrupole time-of-flight mass spectrometer (Agilent Technologies, Santa Clara, USA). The analysis of metabolites from one-carbon metabolism was performed with an ultrahigh-pressure liquid chromatography-quadrupole time-of-flight mass spectrometer (Agilent Technologies). Liquid chromatography was also used in separate analyses to quantify the amounts of guanine, 5-methylcytosine and 5-hydroxymethylcytosine.

DNA and RNA isolation

DNA and RNA were isolated according to the instructions of the Qiagen QIAmp DNA Micro Kit and the Qiagen RNeasy Lipid Tissue Mini Kit (Werfen, Barcelona, Spain), respectively. The concentrations of DNA and RNA were determined using a NanoDrop ND-1000 spectrophotometer (Nanodrop Technologies Inc., Wilmington, NC). The RNA integrity number (score > 8) was assessed using an RNA2100 Bioanalyser (Agilent Technologies, Santa Clara, CA) with the RNA 6000 Nano Kit.

Quantitative real-time PCR

RNA was reverse-transcribed into cDNA using the Reverse Transcription System (Applied Biosystems, Foster City, CA). Quantitative gene expression analyses were conducted on a 7900HT Fast Real-Time PCR System using the reagents provided by the respective TaqMan® Gene Expression Assays (Applied Biosystems) indicated in Table S8. All the measurements were normalised to the expression of the housekeeping gene 18S using standard procedures.

Microarray-based DNA methylation analysis

The samples included in the study were fresh-frozen human livers. DNA extraction, bisulfite conversion, site-specific oxidation and array hybridisation to the 850K microarrays was performed with Infinium human methylation EPIC beadchip array technology according to the manufacturer's instructions (Illumina, San Diego, CA).

Microarray-based gene expression analysis

One hundred nanograms of total RNA purified from the livers was used to produce cyanine 3-CTP-labeled cRNA using the Quick Amp Labeling kit, and 3 µg of labelled cRNA was hybridised to the Sureprint G3 human gene expression 8x60k v2 microarray according to the manufacturer's protocol (Agilent, Palo Alto, CA). The arrays were scanned in an Agilent Microarray Scanner, and the raw data were extracted using Agilent Feature Extraction 10.7.3.1.

Cell culture and flow cytometry analysis

HepG2 cells were obtained from ATTC and grown in high-glucose DMEM (GIBCO) supplemented with 10% fetal bovine serum, 2 mM glutamine, 1% penicillin and streptomycin and nonessential amino acids at 37°C in a humidified atmosphere with 5% CO2. The experiments were performed by adding permeable α-KG (dimethyl-α-ketoglutarate; DMKG) at final concentrations ranging from 0.1 to 2 mM for 72 h and/or metformin at a final concentration of 10 mM. To estimate the percentage of cell death, the cells were detached and mixed with trypan blue to be analysed using the TC20 automated cell counter (Bio-Rad) according to the manufacturer's instructions. After the respective treatments, the cells were either washed two times with phosphate-buffered saline (PBS) and stored at -80°C until the extraction and quantification of metabolites or, for immunoblot assays, the media were replaced with RIPA buffer containing protease and phosphatase inhibitors (P8340, P0044 from Sigma). When required, the cells were stained with annexin V and

propidium iodide for analysis using a BDFACS Canto BD-Biosciences flow cytometer, and the data were assessed using the free software Flowing.

Primary hepatocyte isolation

Primary hepatocytes were isolated from the livers of 5-month-old wild type C57BL/6 J male mice as described.[4] In brief, livers were perfused with Hank's Balanced Salt Solution (Invitrogen) containing 5 mM glucose supplemented with 0.5 mM EGTA and 25 mM HEPES (pH 7.4 at 37°C) using a CTP100 peristaltic pump (ThermoFisher). After exsanguination of the liver, perfusion was changed to DMEM (Sigma-Aldrich D5546) supplemented with 100 U/mL Penicillin and 0.1 mg/mL Streptomycin (Pen/Strep), 15 mM HEPES, and 100 U/mL of collagenase (Type IV, Worthington). Then cells were liberated and cell suspension was then filtered through a 70 µM cell strainer and centrifuged at 50 g x 2 min 3 times. Following centrifugation, cell pellets were resuspended in DMEM (Sigma-Aldrich D5796) supplemented with Pen/Strep, 5 mM HEPES, 10 nM dexamethasone, and seeded into 6-well plates or 24-well Seahorse plates precoated with collagen I (Sigma-Aldrich). One hour later the media was changed to DMEM (Sigma-Aldrich D5546) supplemented with Pen/Strep, 5 mM HEPES, 10 nM dexamethasone and 10% FBS. Media was changed 3 h later to serum-free Earl´s balanced salts (Sigma-Aldrich E2888) supplemented with Pen/Strep, 5 mM HEPES, 10 nM dexamethasone, glucose (1,75 g/l) and sodium pyruvate (1mM), treated or not with metformin and/or DMKG. Cells were cultured for 38 hours for various experiments. After 38 hours of culture, hepatocytes were frozen in liquid nitrogen and lysed in RIA assay buffer (20 mM Tris–HCl (pH 7.5), 150 mM NaCl, 1 mM Na2EDTA, 1 mM EGTA, 1% NP-40, 1% sodium deoxycholate) with protease and phosphatase inhibitors P0044, P5725 and P8340.

Oxygen consumption and ECAR

Mitochondrial bioenergetics of primary hepatocytes was measured using an XF24 Extracellular Flux Analyzer (Agilent).[5] After 38 hours of culture, cells were washed with Seahorse assay media (Seahorse Bioscience). Plates were incubated in a CO_2-free incubator at 37 °C for 1 h to allow temperature and pH equilibration, after which oxygen consumption rate (OCR) was measured in the XF24 Extracellular Flux Analyzer over a period of 88 min. Mitochondrial processes were examined through sequential injections of oligomycin (4 µM) at min 27, carbonyl cyanide 4-(trifluoromethoxy) phenylhydrazone (FCCP; 2 µM) at min 52, rotenone (1µM) at min 78, and antimycin A (5 µM) at min 104. ECAR was determined under basal culture conditions.

Bioinformatics

To analyse the gene expression microarray, spot signals were normalised with Agilent Gene Spring GX v14.8 software and filtered to keep only the autosomal genes for further comparisons (20,214 genes) in R. Using the t.test function, we determined significance of expression difference between NASH and Non-NASH patients (n = 8 each). We determined 345 genes to have $p < 0{\cdot}05$ and $\log_2$ (NASH/Non-NASH) > 1. All RNA expression data were used for the integration of DNA methylation and gene expression data (see below). For the DNA methylation array, the raw data were analysed using the minfi package in R with annotations from Illumina Human Methylation EPICmanifest v0.3.0 and Illumina Human Methylation EPICanno.ilm10b2.hg19 v0.6.0. The data were normalised and filtered to keep only the autosomal CpGs using the preprocess Quantile function with mergeManifest=T and dropLociWithSnps() with snps=c("SBE", "CpG", "Probe") and maf=0 parameters. Each CpG has a beta value (calculated using getBeta), which is the fraction of methylated / methylated + unmethylated signal, bound between 0 and 1. We performed Wilcoxon rank-sum tests on each CpG (n = 8 for each group) to determine significant differences. We also determined the average beta level in NASH and Non-NASH patients and then retained the CpGs whose average Δ beta > 0.05 and whose p-value < 0·05 as significantly differentially methylated. Subsets of CpGs across features of the genome were annotated using the genomation package with hg19 knownGene files downloaded from the UCSC Table Browser, as described. The gplots package was used for heatmap visualisation. To assess the relationship between CpG methylation and gene expression, we merged the differentially methylated CpGs in gene promoters with the RNA microarray data and correlated beta and expression values. *Circos* visualization

was generated using the *circlize* package. Kyoto Encyclopedia of Genes and Genome (KEGG) pathway analysis of selected genes subsets was performed.

References

1. Aranda N, Viteri FE, Montserrat C, Arija V. Effects of C282Y, H63D, and S65C HFE gene mutations, diet, and life-style factors on iron status in a general Mediterranean population from Tarragona, Spain. *Ann Hematol* 2010; **89:** 767–73
2. Mechanick JI, Apovian C, Brethauer S, Garvey WT, Joffe AM, Kim J, et al. Clinical practice guidelines for the perioperative nutrition, metabolic, and nonsurgical support of patients undergoing bariatric procedures. *Surg Obes Relat Dis* 2020; **16:** 175–247
3. Vives M, Molina A, Danús M, Rebenaque E, Blanco S, París M, et al. Analysis of gastric physiology after laparoscopic sleeve gastrectomy (LSG) with or without antral preservation in relation to metabolic response: a randomised study. *Obes Surg* 2017; **27:** 2836–2844
4. Zhang W, Sargis RM, Volden PA, Carmean CM, Sun XJ, Brady MJ. PCB 126 and other dioxin-like PCBs specifically suppress hepatic PEPCK expression via the aryl hydrocarbon receptor. *PLoS One* 2012; **7:** e37103.
5. Qiao A, Jin X, Pang J, Moskophidis D, Mivechi NF. The transcriptional regulator of the chaperone response HSF1 controls hepatic bioenergetics and protein homeostasis. *J Cell Biol* 2017; **216:**723–741
